# Mass spectrometry sequencing of long digital polymers facilitated by programmed inter-byte fragmentation

**DOI:** 10.1038/s41467-017-01104-3

**Published:** 2017-10-17

**Authors:** Abdelaziz Al Ouahabi, Jean-Arthur Amalian, Laurence Charles, Jean-François Lutz

**Affiliations:** 1 Université de Strasbourg, CNRS, Institut Charles Sadron UPR22, 23 rue du Loess, 67034 Strasbourg, France; 2 Aix-Marseille Université, CNRS, UMR 7273, Institute of Radical Chemistry, 13397 Marseille, France

## Abstract

In the context of data storage miniaturization, it was recently shown that digital information can be stored in the monomer sequences of non-natural macromolecules. However, the sequencing of such digital polymers is currently limited to short chains. Here, we report that intact multi-byte digital polymers can be sequenced in a moderate resolution mass spectrometer and that full sequence coverage can be attained without requiring pre-analysis digestion or the help of sequence databases. In order to do so, the polymers are designed to undergo controlled fragmentations in collision-induced dissociation conditions. Each byte of the sequence is labeled by an identification tag and a weak alkoxyamine group is placed between 2 bytes. As a consequence of this design, the NO-C bonds break first upon collisional activation, thus leading to a pattern of mass tag-shifted intact bytes. Afterwards, each byte is individually sequenced in pseudo-MS^3^ conditions and the whole sequence is found.

## Abstract (web summary)

Digital information can be stored in monomer sequences of non-natural macromolecules, but only short chains can be read. Here the authors show long multi-byte digital polymers sequenced in a moderate resolution mass spectrometer. Full sequence coverage can be attained without pre-analysis digestion or the help from sequence databases.

## Introduction

It has been demonstrated in recent years that digital information can be stored in biological^1^ and synthetic macromolecules^2^. In such digital polymers, the monomer units that constitute the chains are used as molecular bits and assembled through controlled synthesis into readable digital sequences^3^. For example, it has been reported that ordered oligonucleotide sequences enable storage of several kilobytes of data in DNA chains^4–7^. Alternatively, our group has demonstrated that binary messages can also efficiently be stored in different types of synthetic macromolecules^8–10^. Overall, molecular encryption in polymers opens up interesting avenues for massive information storage^1^ as well as long-term data storage^11^. Importantly, the use of digitally encoded polymers allows room temperature storage and already gives access to substantial storage capacities. However, the development of practical technologies is currently still limited by relatively slow writing and reading speeds^1^. Various sequencing methods, including tandem mass spectrometry (MS/MS), enzyme-assisted approaches, and nanopore threading, can be used to decipher the coded sequences of biopolymers and man-made macromolecules^12–15^. For biopolymer sequencing, however, the molecular structure of the analyte is fixed by biology and therefore quicker analysis can only be attained through the development of advanced analytical methods. The use of synthetic digital polymers offers an alternative scenario, which is that the molecular structure of the polymer can be tuned to facilitate sequencing using routine analytical instruments^16^.

Here, we report that MS sequencing of long-coded polymer chains can be achieved through careful macromolecular design. Poly(phosphodiester)s chains containing several bytes of information were synthesized and sequenced. Two important molecular features are implemented in the analyte design: alkoxyamine groups are placed between the bytes and each byte is labeled by a mass tag. In collision-induced dissociation (CID) conditions, the weak NO-C bonds are selectively cleaved, thus leading to a series of mass tag-shifted intact bytes. Afterwards, each byte can be individually activated and easily deciphered by MS^3^. Consequently, full sequence coverage can be obtained in a single measurement performed in a moderate resolution mass spectrometer.

## Results

### Design and synthesis of the digital polymers

**Fig. 1:**
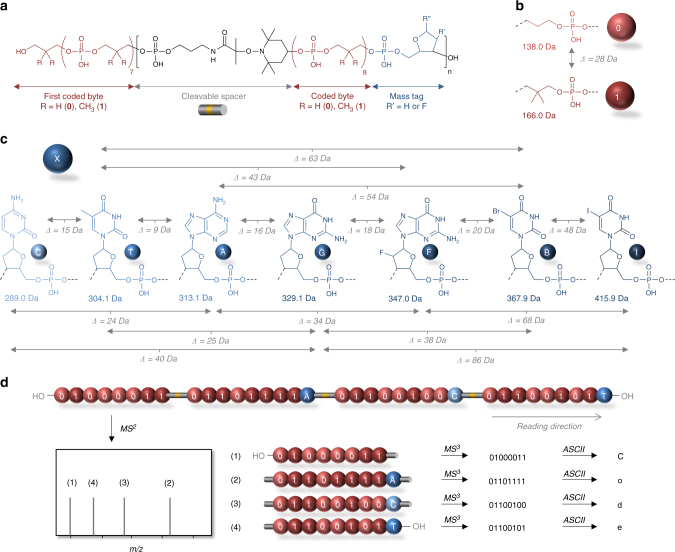
General concept studied herein for the sequencing of long digital polymer chains. **a** Molecular structure of the sequence-coded polymers prepared by automated phosphoramidite chemistry. These digital polymers contain *n* + 1 coded bytes noted in red. A byte is a sequence of eight coded monomers that represent 8 bits. Two consecutive bytes are separated by a linker noted in black, which contains a NO-C bond that can be preferentially cleaved during MS/MS analysis. In order to sort out the bytes after MS/MS cleavage, *n* bytes of the sequence are labeled with a mass tag noted in blue. **b** Molecular structure and mass of the two coded synthons that define the binary code in the polymers. **c** Molecular structure and mass of the mass tags that are used as bytes labels. In order to induce identifiable mass shifts after MS/MS cleavage, the mass of a byte tag (noted in blue) shall not be a multiple of 28, which is the mass difference between a 0 and a 1 coded unit. In addition, the mass difference between two tags (noted in grey) shall not be a multiple of 28. **d** Schematic representation of the mass spectrometry sequencing of a digital polymer containing 4 bytes of information. The polymer is first analyzed in MS/MS conditions, which lead to the favored cleavage of the weak NO-C bonds (depicted in yellow inside the grey spacers). Since they carry mass tags, the resulting cleaved bytes are sorted out by mass in the MS/MS spectrum (the displayed MS^2^ cartoon is idealized for clarity). Afterwards, each byte can be easily sequenced in MS^3^ conditions and the whole binary sequence can be deciphered

We have recently reported that non-natural digital oligomers constructed with repeating units containing alkoxyamine^9, 17^ or carbamate^10^ linkages are very easy to sequence by MS/MS due to the low-energy fragmentation of these bonds. However, in these macromolecules, bond cleavages occur between each bit, which is valid for single-byte oligomers^17, 18^ but might become much more challenging for multi-byte polymers. To favor the sequencing of long digital chains while avoiding complexity issues commonly associated with MS/MS of highly charged chains, we propose in the present work to use a two-stage controlled fragmentation strategy, in which weak bonds are placed between each byte (Fig. 1a) instead of each bit. This concept was applied here to digital poly(phosphodiester)s^8, 19^ that are synthesized by automated phosphoramidite chemistry^20–22^. In such polymers, digital information is written using two monomers that contain either a propyl phosphate (**0**) or a 2,2-dimethylpropyl phosphate (**1**) synthon, as shown in Fig. 1b ^8, 19^. Although multi-bytes chains can be synthesized relatively easily^19^, MS/MS sequencing of long poly(phosphodiester)s containing only repeating phosphate linkages is tedious, as illustrated in Supplementary Fig. 1 by the spectrum of a 4-byte polymer (Supplementary Table 1, Entry 1). However, this spectral complexity is mainly due to an efficient cleavage of all phosphate bonds, leading to eight fragment series that can all be usefully employed for full sequence coverage, in great contrast to MS/MS of DNA, which mostly generate w-type ions as well as numerous useless secondary fragments (Supplementary Fig. 2). To simplify this situation, cleavable NO-C bonds were incorporated in between each byte in the present work. The concept relies on the fact that a NO-C bond requires less energy than a phosphate bond to be broken in CID conditions. Thus, as schematized in Fig. 1d, inter-byte NO-C bonds shall break first during the first activation stage and lead to a MS^2^ spectrum containing all intact individual bytes. Then, subjecting each byte to a second activation stage should yield MS^3^ spectra that would allow an easier sequencing task, thanks to the small size and charge state of dissociating species. However, since bytes may be isobaric (i.e., contain the same number of **0** and **1** units), each byte shall be first labeled with a tag, which allows its identification in MS^2^ and permits to know its location in the initial sequence. Although a wide variety of molecules could be potentially used as byte tags, natural (noted A, T, G, C) and non-natural (noted B, I, F) nucleotides were selected in the present work (Fig. 1c), because the corresponding phosphoramidite monomers are commercially available. As shown in Fig. 1c, the molecular structure of each byte tag was selected in order to create an unequivocal mass and isotopic signature for each byte. In particular, two simple criteria shall be fulfilled: the molar mass of a given byte tag shall not be a multiple of 28, which is the mass difference between a **0** and **1** synthon, and the mass difference between 2-byte tags shall not be a multiple of 28 and shall also not be smaller than 3 Da since triply charged species (vide infra) are studied in MS^3^. The byte tag sequence, T, C, A, G, B, I, F, no tag, was chosen to label the bytes and reading was arbitrarily started from the non-marked byte (i.e., opposite to the sense of synthesis). This means, for example, that the first, penultimate, and last bytes of a sequence always contain no tag, a C-tag, and a T-tag, respectively. Hence, after performing MS, MS^2^, and MS^3^ steps, the whole sequence of the original polymer can be comprehensively reconstructed.

In order to verify the feasibility of this concept, model polymers containing only 2 bytes of information separated by one single cleavable NO-C site were first studied (Supplementary Table 1, Entries 3, 4). In particular, two different alkoxyamines containing either a mono- or a di-methylated carbon were considered. These cleavable groups were incorporated in between the bytes during polymer synthesis using phosphoramidite monomers **a1** and **a2** (Supplementary Fig. 3). It was found that the mono-methylated alkoxyamine **a1** is not optimal because the energy threshold for the NO-C bond homolysis appears to be close to that of inner-byte phosphate bond cleavage, thus resulting in a polluted MS^2^ spectrum (Supplementary Fig. 4a). On the other hand, the more labile NO-C linkage in di-methylated **a2** leads to preferential fragmentation in MS^2^ conditions, where phosphate bonds do not break (Supplementary Fig. 4b). Yet, it should be remarked that **a2** leads to some slight in-source byte fragmentation in the initial MS analysis of the complete polymer. However, these ions can be easily tracked and disclosed based on their much lower charge state compared to the intact macromolecule. Thus, alkoxyamine **a2** was selected for the synthesis of digital poly(phosphodiester)s containing 4, 5, 6, or 8 bytes of information (Supplementary Table 1). All these polymers were synthesized by automated phosphoramidite chemistry. For each monomer attachment, cycles involving dimethoxytrityl (DMT) deprotection of the reactive alcohol sites; phosphoramidite coupling; oxidation of the formed phosphite into a phosphate; and capping of the unreacted alcohol sites by reaction with acetic anhydride were used. Although the latter capping step is not mandatory for the synthesis of short oligomers, it is crucial for the synthesis and purification of long sequence-defined polymers^19^. After synthesis, the DMT-terminated digital sequences were cleaved from the support and purified on a reverse-phase cartridge. This purification process allows separation of the desired DMT-terminated sequences from failure sequences capped by acetic anhydride^19^. Afterwards, the terminal DMT group is removed. As shown in Supplementary Table 1, all polymers were obtained in good yields after reverse-phase column purification.

### Mass spectrometry sequencing of the multi-byte polymers

**Fig. 2:**
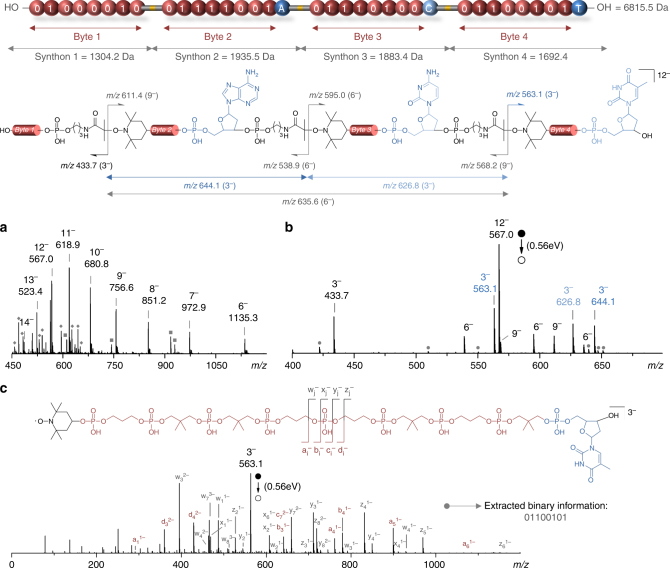
Sequencing of a 4-byte digital polymer that contains the ASCII-encoded word Byte. **a** High-resolution electrospray mass spectrum (MS^1^) obtained in the negative ion mode for a 4-byte digital polymer (Supplementary Table 1, Entry 5). The upper numbers represent the different charge states observed for the polymer. Dark grey diamonds indicate in-source fragments (see Supplementary Table 2 for a detailed interpretation of each peak) and dark grey squares designate different charge states of two different synthesis impurities. **b** MS^2^ spectrum (0.56 eV, center of mass frame) obtained by collision-induced dissociation of the [M-12H]^12−^ precursor ion, which carries on average three charges per byte. In this case, the MS/MS single-byte, double-byte, and triple-byte fragments are predominantly observed as trianions, hexa-anions, and nona-anions, respectively. Other charge states can be observed to a minor extent and are denoted by dark grey circles (see Supplementary Table 2 for a detailed interpretation of each peak). **c** Molecular sequencing (pseudo-MS^3^) of a byte fragment obtained by collision-induced dissociation (0.56 eV, center of mass frame) of the precursor trianion [M-3H]^3−^. For clarity, only the sequencing of byte 4 is shown as an example in this figure. The sequencing of bytes 1–3 is shown in Supplementary Fig. 6

Figure 2 shows the three-stage analysis of a polymer containing 4 bytes of information (Supplementary Table 1, Entry 5). This polymer was first analyzed by negative mode electrospray ionization (ESI)-MS, which revealed a dominant charge state distribution (Fig. 2a). Among all detected polyanions, the dodeca-anion [M-12H]^12−^ was selected for MS^2^: because it contains, on average, three deprotonated phosphate groups per byte, its dissociation leads to byte fragments at a preferential −3 charge state. It should be specified that MS^2^ experiments were not performed here for sequencing purposes, and traditionally defined as the production of fragments that differ in mass by a single building unit and hence allow the original chain to be reconstructed. Instead, activation of precursor ions aims here at producing fragments that all contain a single byte (to be further sequenced in MS^3^). However, since dissociation of precursor with *n* bytes proceeds by competitive NO-C bond cleavages, primary product ions contain from 1 byte (either the first or the last one) to *n*−1 bytes. Collision energy was hence raised to promote consecutive dissociations of large primary product ions, in order to form secondary fragments that each contains one inner-chain byte. Energy has, however, to remain below dissociation threshold of phosphate bonds to prevent inner-byte fragmentation. In such conditions, a very clear bytes pattern can be observed in the resulting MS^2^ spectrum (Fig. 2b). Each byte appears predominantly as a trianion (while remaining as minor signals at −2 or −4 charge states) and the byte tags lead to unambiguous mass shifts that allow identification of their initial location in the chain (Supplementary Table 3). Importantly, the concept also works for polymers containing similar or isomeric bytes. For instance, Supplementary Fig. 5 shows the sequencing of a polymer containing four times the same byte (Supplementary Table 1, Entry 6). Even in such a case, the byte tags allow unequivocal detection of each byte in MS^2^. Fragments resulting from partially cleaved chains, hence containing either two or three bytes and, respectively, detected at −6 and −9 charge states, were also observed in MS/MS spectra, as exemplified in Fig. 2b. After MS^2^ byte cleavage, each byte was individually sequenced by MS^3^. In order to take advantage of the resolving capabilities offered by orthogonal acceleration time-of-flight (oa-TOF) mass analyzers to safely assign fragments, the Q-oa-TOF instrument used here for MS and MS^2^ stages was also employed to perform pseudo-MS^3^ experiments. Typically, deprotonated polymers were first activated in the instrument interface by raising the cone (or skimmer) voltage to perform in-source CID; then, so-released byte fragments were mass selected in the quadrupole for further excitation in the collision cell and fragment measurement in the oa-TOF. Figure 2c shows, for example, the sequencing of the T-tagged last byte of polymer **5** and the sequencing of bytes 1–3 is shown in Supplementary Fig. 6. Hence, the complete digital sequence of the polymer was easily deciphered. In order to evidence the universality of this concept, polymers with other 4-byte sequences (Supplementary Table 1, Entries 6, 7) were analyzed (Supplementary Figs. 7–11).

**Fig. 3:**
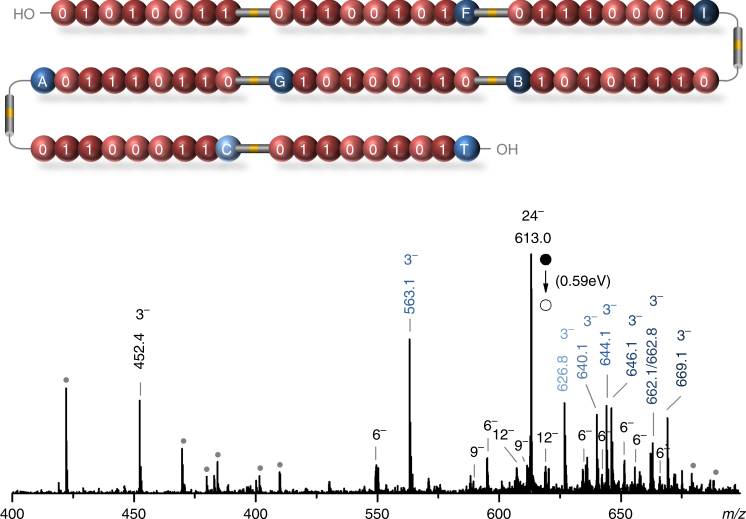
MS^2^ spectrum of an 8-byte digital polymer containing the ASCII-encoded word Sequence. This spectrum (0.59 eV, center of mass frame) was obtained by collision-induced dissociation of the [M-24H]^24−^ precursor ion of polymer 11 in Supplementary Table 1. In this case, the MS/MS single-byte, double-byte, triple-byte, and tetra-byte fragments are predominantly observed as trianions, hexa-anions, nona-anions, and dodeca-anions, respectively. Other charge states can be observed to a minor extent and are denoted by dark grey circles (see Supplementary Figs. 21b and 22 for a detailed interpretation of each peak). The subsequent pseudo-MS^3^ sequencing of all bytes is shown in Supplementary Figs. 23–25

Supplementary Figs. 12–20 show the data obtained for 5- and 6-byte polymers (Supplementary Table 1, Entries 8–10) and confirm that messages encoded in longer polymers can also be easily deciphered using the proposed multi-step sequencing. It is, however, interesting to point out that 5-byte polymers were only labeled with natural byte tags A, T, G, C, whereas the 6-byte polymer also contains a non-natural byte tag B. The latter contains a bromine atom and, therefore, does not only allow byte identification by mass but also by isotopic pattern. Ultimately, an 8-byte polymer (Supplementary Table 1, Entry 11) was synthesized and analyzed. Figure 3 shows the MS^2^ spectrum obtained from the precursor anion containing 24 negative charges (i.e., three charges in average per byte). After promoting multiple in-chain NO-C fragmentations, a clear pattern of byte trianions was measured in MS^2^, thus allowing individual byte sequencing by pseudo-MS^3^ (Supplementary Figs. 21–25).

## Discussion

The development of practical polymer-based digital memories requires libraries of macromolecules that contain at least a few bytes of data in each chain^1^. Sequence-coded polymer chains containing more than a hundred bits can be synthesized using automated solid-phase chemistry^19^ or inkjet technologies^23^. However, MS sequencing of such long chains is very challenging, in particular, when targeting full sequence coverage as mandatory for digital polymers. Indeed, assuming an efficient collisional activation, the total ion current is spread over a large number of fragments. Moreover, these fragments are most often produced at different charge states because they are formed from highly charged macromolecules generated by ESI, a soft technique used to ensure their structural integrity. For instance, when full sequence coverage is required, it is known that MS/MS is most efficient for the sequencing of peptides containing less than 20 residues and that the analysis of longer proteins requires enzymatic digestion and HPLC separation of complex mixtures^24^. Similarly, MS/MS sequencing of intact nucleic acids is not trivial^25^. Such a situation can hardly be improved, as the molecular structure of biopolymers is set by biology.

In contrast, as demonstrated in this article, synthetic polymer chemistry allows design of digital polymers that may undergo controlled fragmentations in MS/MS conditions. Indeed, the experimental data highlighted in this paper indicate that intact long sequence-coded chains can be fully sequenced in a routine mass spectrometer operating at low collision energy, without requiring digestion, purification, or separation steps prior to analysis. The use of databases is also not mandatory in this approach to decipher the coded sequences. To attain such a MS readability, the molecular structure of the polymers was carefully engineered and, in particular, two key features were implemented: the use of cleavable inter-bytes spacer that promotes programmed MS^2^ fragmentation and the use of mass tags that allow identification of byte original location. The former feature allows the actual MS sequencing task to be limited to very short (8 bits) oligo(phosphodiester)s, while the latter one permits reliable reconstruction of the byte sequence. It should be noted that the reported concept is not limited to poly(phosphodiester)s and could be extended to other types of digital polymers. For instance, alkoxyamine groups are most probably not the only type of inter-byte links that can be used in this approach. Depending on the type of digital polymer that shall be deciphered by MS, other cleavable linkers may be imagined. In fact, the general rule is that the inter-byte linkers require less energy for being broken in CID conditions than the intra-byte bonds that connect the bits. Furthermore, byte tags shall not necessarily be nucleotides. As mentioned in the results section, these markers have been selected in the present work because their phosphoramidite derivatives are commercially available. Yet, other byte tags can be envisioned. Here, the general rule is that the molar mass of a byte tag and the mass difference between two byte tags shall not be a multiple of the mass difference between 2 bits. In addition, markers with markedly different isotopic signatures can also be imagined.

Overall, this work opens up interesting perspectives for the design of polymer-based molecular memories^1^. For such technological applications, it is important to specify that the synthesis of very-long-digital polymers (i.e., linear chains containing several hundreds of coded bits) is not an objective. Indeed, polymer-based memory devices will most probably rely on libraries of coded chains, as already done in the field of DNA storage^4, 5^. In such libraries, individual chains containing about 100 coded residues and a short localization address sequence are typically used and permit to store large quantities of information^4^. In addition, coding theory^7^ and enhanced monomer alphabets^16^ can be used to increase storage density in short segments. In this context, the results of the present paper underline that an important milestone in terms of chain length has now been reached for synthetic digital polymers. Indeed, it is now possible to encode and comprehensively decode long non-natural chains, as demonstrated in this work with a chain containing 78 residues (64 bits, 7 tags, and 7 spacers). The next important challenge in the field of polymer-based data storage will, therefore, probably be the development of organized and accessible digital polymers libraries.

## Methods

### Materials

2-cyanoethyl diisopropyl-chlorophosphoramidite (95%, Alfa Aesar), 4,4′-dimethoxytriphenylmethyl chloride (≥97.0%, Sigma-Aldrich), *N*,*N*-diisopropylethylamine (DIPEA, 99%, Alfa Aesar), triethylamine (99%, Alfa Aesar), 1,1,4,7,7-pentamethyldiethylenetriamine (PMDTA, 98%, Alfa Aesar), 3-amino-1-propanol (99%, Alfa Aesar), anhydrous methylamine (AMA, 98%, Sigma-Aldrich), ammonium hydroxide solution (28.0–30.0% NH_3_, Sigma-Aldrich), 2-bromopropionyl bromide (97%, Sigma-Aldrich), α-bromoisobutyryl bromide (98%, Sigma-Aldrich), 4-hydroxy-TEMPO (97%, Sigma-Aldrich), and copper(I) chloride (97%, Sigma-Aldrich) were used as purchased. Anhydrous dichloromethane (DCM), pyridine, and acetonitrile were purchased from Sigma-Aldrich. Anhydrous THF was obtained using a dry solvent station GT S100. All air-sensitive reactions have been carried out under argon atmosphere. Automated synthesizer reagents, anhydrous acetonitrile (ACN, Glen Research), oxidizing solution (0.02 M I_2_ in THF/H_2_O/pyridine), deblocking mix (3% TCA in DCM), cap Mix A (THF/Ac_2_O), activator (tetrazole in acetonitrile) were purchased from Glen Research. Nucleoside-functionalized controlled pore glass columns (T-lcaa-CPG), nucleoside phosphoramidites: dT-CE phosphoramidite, Pac-dA-CE phosphoramidite, Ac-dC-CE phosphoramidite, iPr-Pac-dG-CE phosphoramidite, 2′-F-G-CE phosphoramidite, 5-Br-dC-CE phosphoramidite, 5-I-dU-CE phosphoramidite, and PolyPak II reverse-phase cartridges were also obtained from Glen Research. All the phosphoramidites were stored in the freezer at −18 °C.

### Monomer synthesis

Supplementary Fig. 3 shows the molecular structures of the phosphoramidite monomers that were used in this work to synthesize digital polymers. The phosphoramidite monomers **0** and **1** were used as binary coding units and were synthesized as described in a previous publication^8^. The byte tags A, T, C, G, B, F, and I are commercial nucleotides, as described in the previous paragraph. The alkoxyamine-containing monomers **a1** and **a2** were synthesized following the strategy depicted in Supplementary Fig. 26. For all intermediate compounds, the raw ^1^H and ^13^C NMR spectra are shown in Supplementary Figs. 27–33. 2-bromo-*N*-(3-hydroxypropyl)propanamide (**d1**). The compound **d1** was synthesized according to a reported procedure^17^ using 2-bromopropionyl bromide (9.6 g, 44.4 mmol), 3-aminopropan-1-ol (3.3 g, 44.0 mmol) and triethylamine (4.9 g, 47.8 mmol) in 60 ml of anhydrous DCM to give a colorless oil in 82% yield. ^1^H NMR (400 MHz, CDCl_3_, δ, ppm): 6.87 (s, 1H, -NH), 4.42 (q, *J* = 7.0 Hz, 1H, Br-CH-(CH)_3_), 3.67 (t, *J* = 5.5 Hz, 2H, HO-CH_2_-), 3.44 (m, 2H, -NH-CH_2_-CH_2_-), 2.85 (s, 1H, -OH), 1.86 (d, 3H, -CH_3_), 1.74 (m, 2H, -NH-CH_2_-CH_2_-). ^13^C NMR (75 MHz, CDCl_3_, δ, ppm): 170.4, 59.6, 45.0, 37.2, 31.7, 23.2 ppm. High resolution mass spectrometry (HRMS) (*m/z*) [M + H]^+^ calcd. for C_6_H_13_BrNO_2_^+^, 210.0130; found 210.0145. 2-bromo-*N*-(3-hydroxypropyl)-2-methylpropanamide (**d2**). The synthesis of **d2** was described in a previous publication^17^. 2-((4-hydroxy-2,2,6,6-tetramethylpiperidin-1-yl)oxy)-*N*-(3-hydroxypropyl)propanamide (**c1**). To a solution of hydroxy-TEMPO (1.72 g, 10 mmol) in ethyl acetate (10 ml) CuCl (2.0 g, 20 mmol) and **d1** (2.26 g, 10.8 mmol) were added under argon. Then, *N*,*N*,*N*′,*N*′′,*N*′′-pentamethyldiethylenetriamine (PMDTA) (3.46 g, 20 mmol) was added dropwise within 45 min while keeping the temperature at 35–38 °C. The mixture was then stirred for 12 h at room temperature under argon. The green suspension was filtered and the filter cake was washed with 100 ml ethyl acetate. The filtrate was washed successively with 3 × 20 ml water, dried over MgSO_4_, evaporated, and finally chromatographed on silica gel (ethyl acetate) to afford 2.6 g (87%) of **c1** as a colorless oil leading later to a white crystalline powder. TLC (ethyl acetate): Rf = 0.50; ^1^H NMR (400 MHz, CDCl_3_, δ, ppm): 6.72 (s, 1H, -NH), 4.31 (m, 1H, -NO-CH(CH_3_)-), 3.95 (m, 1H, HO-CH-), 3.63 (t, *J* = 5.0 Hz, 2H, HO-CH_2_-), 3.45 (m, 2H, -NH-CH_2_-CH_2_-), 3.12 (s, 1H, -OH), 1.78–1.85 (m, 2H, -N-(C(CH_3_)_2_)_2_-CH_2_-), 1.72 (m, 2H, -NH-CH_2_-CH_2_-), 1.45–1.47 (d, 3H, -NO-CH(CH_3_)-), 1.40 (m, 2H, -N-(C(CH_3_)_2_)_2_-CH_2_-), 1.15–1.22 (m, 12H, -N-(C-(CH_3_)_2_)_2_). ^13^C NMR (75 MHz, CDCl_3_, δ, ppm): 175.0, 82.3, 62.9, 60.7, 59.7, 59.1, 48.5, 35.5, 32.4, 21.5, 19.5 ppm. HRMS (*m/z*) [M + H]^+^ calcd. for C_15_H_31_N_2_O_4_^+^, 303.2206; found 303.2279. 2-((4-hydroxy-2,2,6,6-tetramethylpiperidin-1-yl)oxy)-*N*-(3-hydroxypropyl)-2-methylpropanamide (**c2**). The compound **c2** was isolated in 90% yield following the same procedure for **c1** using **d2** (2.42 g, 10.8 mmol), hydroxy-TEMPO (1.72 g, 10 mmol), CuCl (2.0 g, 20 mmol) and PMDTA (3.46 g, 20 mmol). TLC (ethyl acetate): Rf = 0.48; ^1^H NMR (400 MHz, CDCl_3_, δ, ppm): 6.79 (s, 1H, -NH), 3.95 (m, 1H, HO-CH-), 3.61 (t, *J* = 4.9 Hz, 2H, HO-CH_2_-), 3.42 (m, 2H, -NH-CH_2_-CH -), 3.19 (s, 1H, -OH), 1.83–1.87 (m, 2H, -N-(C(CH_3_)_2_)_2_-CH_2_-), 1.71 (m, 2H, -NH-CH_2_-CH_2_-), 1.48 (s, 6H, -NO-C(CH_3_)_2_-), 1.46 (m, 2H, -N-(C(CH_3_)_2_)_2_-CH_2_-), 1.11–1.18 (d, *J* = 29.6 Hz, 12H, -N-(C-(CH3)2)2). ^13^C NMR (75 MHz, CDCl_3_, δ, ppm): 176.7, 83.0, 62.8, 61.6, 59.9, 59.1, 49.1, 35.6, 33.5, 32.6, 25.1, 21.6 ppm. HRMS (*m/z*) [M + H]^+^ calcd. for C_16_H_33_N_2_O_4_^+^, 317.2440; found 317.2435. *N*-(3-(bis(4-methoxyphenyl)(phenyl)methoxy)propyl)-2-((4-hydroxy-2,2,6,6-tetramethyl-piperidin-1-yl)oxy)propanamide (**b1**). The compound **c1** (1.96 g, 6.5 mmol) was co-evaporated with 5 ml of anhydrous pyridine. Afterwards, 10 ml of anhydrous pyridine and 12 ml of anhydrous THF were introduced and reacted with 4,4-dimethoxytrityl chloride (DMTCl) (2.22 g, 6.5 mmol). The DMTCl was added in three portions at a rate of one portion every 45 min. After the three additions, the mixture was stirred at room temperature for 2 h. The reaction was stopped with the addition of 5 ml of methanol and the mixture was evaporated to dryness. The residue was dissolved in ethyl acetate (40 ml) and washed with 5% sodium bicarbonate ice-cold solution. The aqueous layer was extracted with 40 ml of ethyl acetate. The combined organic layers were washed with water and brine, dried with anhydrous Na_2_SO_4_ and evaporated. The resulting product was chromatographed on silica gel (50% ethyl acetate in cyclohexane with 1% triethylamine) yielding 3.10 g of **b1** (77%) as a white crystalline powder. TLC (cyclohexane: ethyl acetate: triethylamine, 50:50:1 v/v/v): Rf = 0.36; ^1^H NMR (400 MHz, CDCl_3_, δ, ppm): 7.40 (d, *J* = 7.6 Hz, 2H, -DMT), 7.29 (m, 6H, DMT), 7.25 (m, 1H, DMT), 6.80 (d, *J* = 6.5 Hz, 4H, DMT), 6.41 (m, 1H, -NH), 4.20 (m, 1H, -NO-CH(CH_3_)-), 3.93 (m, 1H, HO-CH-), 3.78 (s, 6H, 2× -O-CH_3_), 3.44 (m, 2H, DMTO-CH_2_-), 3.14 (m, 2H, -NH-CH_2_-CH_2_-), 1.61–1.83 (m, 4H, -NH-CH_2_-CH_2_-, -N-(C(CH_3_)_2_)_2_-CH_2_-), 1.40 (m, 2H, -N-(C(CH_3_)_2_)_2_-CH_2_-), 1.38–1.40 (d, *J* = 6.9 Hz, 3H, -NO-CHCH_3_-), 1.09–1.18 (d, *J* = 18.2 Hz, 12H, -N-(C-(CH_3_)_2_)_2_). ^13^C NMR (75 MHz, CDCl_3_, δ, ppm): 173.6, 158.4, 145.0, 136.3, 129.9, 128.1, 127.8, 126.7, 113.1, 86.0, 82.9, 61.2, 60.5, 59.7, 55.2, 49.1, 36.7, 30.9, 29.9, 19.3 ppm. HRMS (*m/z*) [M + H]^+^ calcd. for C_36_H_49_N_2_O_6_^+^, 605.3585; found 605.3586. *N*-(3-(bis(4-methoxyphenyl)(phenyl)methoxy)propyl)-2-((4-hydroxy-2,2,6,6-tetramethylpiperidin-1-yl)oxy)-2-methylpropanamide (**b2**). The compound **b2** was isolated in 80 % yield as a white crystalline powder following the same procedure for **b1** starting from **c2** (2.06 g, 6.5 mmol) and 4,4-dimethoxytrityl chloride (DMTCl) (2.22 g, 6.5 mmol). TLC (cyclohexane: ethyl acetate, 6:4 v/v): Rf = 0.40; ^1^H NMR (400 MHz, CDCl_3_, δ, ppm): 7.41 (d, *J* = 7.1 Hz, 2H, DMT), 7.30 (m, 6H, DMT), 7.20 (m, 1H, DMT), 6.80–6.82 (d, *J* = 8.9 Hz 4H, DMT), 6.51 (s, 1H, -NH), 3.94 (m, 1H, HO-CH-), 3.78 (s, 6H, 2× -O-CH_3_), 3.42 (m, 2H, DMTO-CH_2_-), 3.13 (t, *J* = 6.1 Hz, 2H, -NH-CH_2_-CH_2_-), 1.81 (m, 4H, -NH-CH_2_-CH_2_-, -N-(C(CH_3_)_2_)_2_-CH_2_-), 1.42 (m, 2H, -N-(C(CH_3_)_2_)_2_-CH_2_-; s, 6H, -NO-C(CH_3_)_2_-), 1.07–1.11 (d, *J* = 18.3 Hz, 12H, -N-(C-(CH_3_)_2_)_2_). ^13^C NMR (75 MHz, CDCl_3_, δ, ppm): 176.5, 158.3, 144.9, 136.2, 129.9, 128.1, 127.7, 126.6, 113.0, 85.9, 83.1, 62.8, 61.0, 59.9, 55.1, 49.0, 36.8, 33.3, 29.9, 26.8, 24.9, 21.6 ppm. HRMS (*m/z*) [M + H]^+^ calcd. for C_37_H_51_N_2_O_6_^+^, 619.3669; found 619.3734. 1-((1-((3-(bis(4-methoxyphenyl)(phenyl)-methoxy)propyl)amino)-1-oxopropan-2-yl)oxy)-2,2,6,6-tetramethylpiperidin-4-yl (2-cyanoethyl) diisopropylphosphoramidite (**a1**). The compound **b1** (2.5 g, 4.2 mmol) was co-evaporated with 10 ml of anhydrous DCM and dried under vacuum over 30 min, then 0.1 g of molecular sieves 3 Å were added. About 10 ml of anhydrous DCM and DIPEA (3 ml, 17 mmol) were added successively under argon and the mixture was cooled at 0 °C. *O*-2-cyanoethyl-*N,N*-diisopropyl-chlorophosphoramidite (0.95 ml, 4.2 mmol) was added dropwise with continuous stirring. The reaction flask was then allowed to warm to room temperature and stirred for 1 h. The mixture was concentrated and diluted with ethyl acetate then chromatographed on silica gel (50% ethyl acetate in cyclohexane with a 1% triethylamine) yielding 2.4 g of **a1** (95%) as a white crystalline powder. TLC (cyclohexane: ethyl acetate: triethylamine, 50:50:1 v/v/v): Rf = 0.37; ^31^P NMR (161.92 MHz, CDCl_3_, δ, ppm): 145.37. ^1^H NMR (400 MHz, CDCl_3_, δ, ppm): 7.41 (d, *J* = 7.3 Hz, 2H, DMT), 7.29 (m, 6H, DMT), 7.19 (m, 1H, DMT), 6.80–6.82 (d, *J* = 8.7 Hz, 4H, DMT), 6.42 (s, 1H, -NH), 4.20 (m, 1H, -NO-CH(CH_3_)-), 4.05 (m, 1H, PO-CH-), 3.81–3.83 (m, 2H, NC-CH_2_-CH_2_-O-P), 3.78 (s, 6H, 2× -O-CH_3_), 3.58–3.61 (m, 2H, -N-CH-(CH_3_)_2_), 3.43 (m, 2H, DMTO-CH_2_-), 3.14 (m, 2H, -NH-CH_2_-CH_2_-), 2.62 (t, *J* = 6.4 Hz, 2H, NC-CH_2_-CH_2_-), 1.82–1.84 (m, 4H, -NH-CH_2_-CH_2_-, -N-(C(CH_3_)_2_)_2_-CH_2_-), 1.58 (m, 2H, -N-(C(CH_3_)_2_)_2_-CH_2_-), 1.37 (d, *J* = 6.9 Hz, 3H, -NO-CH(CH_3_)-), 1.17–1.19 (m, 12H, -N-CH-(CH_3_)_2_), 1.08–1.12 (m, 12H, -N-(C-(CH_3_)_2_)_2_). ^13^C NMR (75 MHz, CDCl_3_, δ, ppm): 173.6, 158.3, 144.9, 136.2, 129.9, 128.0, 127.7, 126.6, 117.6, 113.0, 86.0, 82.8, 61.2, 61.0, 60.5, 59.8, 59.7, 55.1, 47.6, 43.0, 42.9, 36.6, 29.8, 24.6, 24.5, 24.4, 20.3, 19.2 ppm. HRMS (*m/z*) [M + Li]^+^ calcd. for C_45_H_65_LiN_4_O_7_P^+^, 811.4745; found 811.4746. 1-((1-((3-(bis(4-methoxyphenyl)(phenyl)methoxy)propyl)amino)-2-methyl-1-oxopropan-2yl)oxy)-2,2,6,6-tetramethylpiperidin-4-yl (2-cyanoethyl) diisopropylphosphoramidite (**a2**). The compound **a2** was isolated in 96 % yield as a white crystalline powder following the same procedure for **a1**, starting from **b2** (2.6 g, 4.2 mmol) and *O*-2-cyanoethyl-*N,N*-diisopropyl-chlorophosphoramidite (0.95 ml, 4.2 mmol). TLC (cyclohexane: ethyl acetate: triethylamine, 50:50:1 v/v/v): Rf = 0.50; ^31^P NMR (161.92 MHz, CDCl_3_, δ, ppm): 145.37. ^1^H NMR (400 MHz, CDCl_3_, δ, ppm): 7.41 (d, *J* = 7.1 Hz, 2H, DMT), 7.29 (m, 6H, DMT), 7.19 (m, 1H, DMT), 6.80–6.82 (d, *J* = 8.9 Hz, 4H, DMT), 6.51 (s, 1H, -NH), 4.02 (m, 1H, PO-CH-), 3.81–3.83 (m, 2H, NC-CH_2_-CH_2_-O-P), 3.78 (s, 6H, 2× -O-CH_3_), 3.58–3.61 (m, 2H, -N-CH-(CH_3_)_2_), 3.42 (m, 2H, DMTO-CH_2_-), 3.13 (m, 2H, -NH-CH_2_-CH_2_-), 2.63 (t, *J* = 6.4 Hz, 2H, NC-CH_2_-CH_2_-), 1.82 (m, 4H, -NH-CH_2_-CH_2_-, -N-(C(CH_3_)_2_)_2_-CH_2_-), 1.56 (m, 2H, -N-(C(CH_3_)_2_)_2_-CH_2_-), 1.41 (s, 6H, -NO-C(CH_3_)_2_-), 1.17–1.19 (m, 12H, -N-CH-(CH_3_)_2_), 1.07–1.11 (m, 12H, -N-(C-(CH_3_)_2_)_2_). ^13^C NMR (75 MHz, CDCl_3_, δ, ppm): 176.4, 158.3, 144.9, 136.2, 129.8, 128.0, 127.7, 126.6, 117.5, 112.9, 85.8, 83.1, 65.1, 64.9, 61.0, 60.0, 58.2, 58.0, 55.1, 47.7, 47.6, 42.8, 36.8, 33.3, 29.8, 24.8, 24.5, 24.3, 21.6, 20.2, 19.8 ppm. HRMS (*m/z*) [M + Li]^+^ calcd. for C_46_H_67_LiN_4_O_7_P^+^, 825.4907; found 825.4872.

### Polymer encoding and automated solid-phase synthesis

All the polymers chains studied in this work were encoded following extended ASCII rules (Supplementary Table 1). The bytes sequences were labeled following the self-defined order T, C, A, G, B, I, F, no tag. The chains were conceived to be read from the terminal non-tagged byte. In other words, the reading direction is opposite to the synthesis direction. All polymers were encrypted following these simple rules and were synthesized using conventional automated solid-phase phosphoramidite method on an Expedite DNA Synthesizer (Perseptive Biosystem 8900). Each cycle involved deprotection, coupling, oxidation, and capping steps, as previously described^19^. The phosphoramidites **0**, **1**, **a1**, **a2**, A, T, C, G, B, I, and F were dissolved in anhydrous acetonitrile under argon (60 mM solution), placed in the synthesizer with all the reagents and primed twice. The solid support-filled column (1 µmol scale) was placed in the synthesizer and the automated syntheses of the different sequences were started with DMT-ON mode. Once the syntheses were finished, the columns were removed from the synthesizer and flushed with argon. The DMT-protected polymers were cleaved from the solid support using a solution of 28% ammonia and methylamine (1/1, v/v) at room temperature for 1 h and purified on a PolyPak II reverse-phase cartridge following a previously reported protocol^19^. After removal of the capped failure sequences, the terminal DMT group of the targeted sequence-coded polymers were cleaved on the column and washed out by solvent elution^19^. The isolated polymers solutions were lyophilized, then weighed and the recovery yields are summarized in Supplementary Table 1. A typical ^1^H NMR spectrum of a purified polymer is shown in Supplementary Fig. 34.

### Measurements and analysis

The ^1^H NMR spectra were recorded in CDCl_3_ on a Bruker Avance 400 MHz spectrometer. The ^13^C NMR spectra were recorded at 100.6 MHz. The ^31^P NMR spectra were recorded at 161.92 MHz and were externally referenced to 85% phosphoric acid. All MS experiments were performed with a Waters Synapt G2 HDMS quadrupole/time-of-flight (QTOF) tandem mass spectrometer (Manchester, UK). All samples were prepared in a methanolic solution of ammonium acetate (3 mM), and introduced at a 10 μl min^−1^ flow rate in the ESI source operated in the negative mode (capillary voltage: −2.27 kV) under a desolvation gas (N_2_) flow of 100 l h^−1^ heated at 35 °C. The cone voltage was −10 or −20 V for both MS and MS^2^ experiments while it was adjusted between −20 and −50 V to induce in-source fragmentation in MS^3^ experiments. CID was performed in the ion trap device using argon as the collision gas after selection of the primary (in MS^2^) or secondary (in MS^3^) precursor ion in the quadrupole mass analyzer of the instrument. Data analyses were conducted using the MassLynx 4.1 program provided by Waters. While fragmentation reactions occurring during the first activation stage (MS^2^ experiments) mainly consist of NO-C bond homolysis, two main types of dissociation pathways were experienced by byte fragments when excited in the second activation stage (pseudo-MS^3^ experiments). The first one comprises all cleavages of backbone bonds of the poly(phosphodiester) chain that give rise to α-containing (i.e., a_i_^z−^, b_i_^z−^, c_i_^z−^, d_i_^z−^) or ω-containing (i.e., w_i_^z−^, x_i_^z−^, y_i_^z−^, z_i_^z−^) fragments typically employed to reconstruct the sequence from the left- or from the right-hand side of the byte, respectively^26^. The second one is due to the reactivity of the carbon-centered radical in the ω-termination of all byte fragments but the last one (Supplementary Fig. 35), and is observed to generate numerous product ions of usually high abundance (annotated in grey in pseudo-MS^3^ spectra). Among these reactions, some are characterized by elimination of a radical species from the activated precursor, as illustrated in Supplementary Fig. 36. Other reactions were observed to proceed via neutral loss, as shown with the elimination of a 225.1 Da species, also observed as a deprotonated molecule at *m/z* 224.1 (Supplementary Fig. 37). Some reactions proceed via the release of species that contain the base tag and can hence confirm its nature (Supplementary Fig. 38). Finally, and as a more useful evidence of the nature of the base use to tag byte fragments, a radical anion that contains the tagging base is systematically observed in the low *m/z* range of pseudo-MS^3^ spectra (Supplementary Fig. 39).

### Data availability

All data are available from the authors upon reasonable request.

## Electronic supplementary material

Supplementary Information
