# Supplementary material for: Mass spectrometry sequencing of long digital polymers facilitated by programmed inter-byte fragmentation
Source: Nat Commun. 2017 Oct 17;8:967. doi: 10.1038/s41467-017-01104-3 (PMC5645402; doi:10.1038/s41467-017-01104-3)
Supplement: Supplementary file 1 — Supplementary Information [file 41467_2017_1104_MOESM1_ESM.pdf]

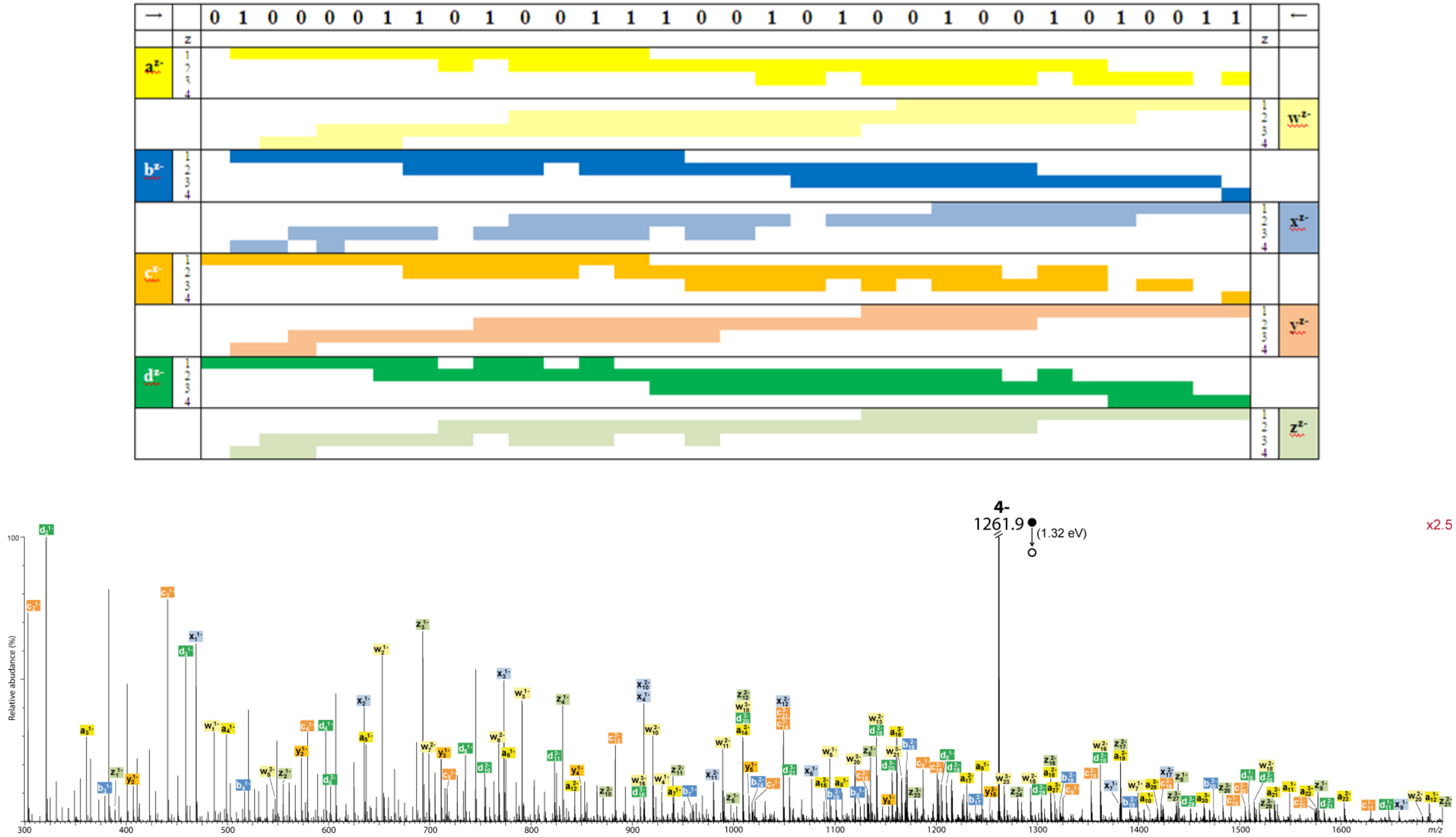

Supplementary Figure 1. ESI-MS/MS of $[M-4H]^{4-}$ at *m/z* 1261.7 for a digitally-encoded poly(phosphodiester) containing 32 bits (4 bytes) of information coding for CNRS (Supplementary Table 1, Entry 1). Top : sequence coverage using each of the eight fragment series. Fragments containing the α termination (a^{z-}, b^{z-}, c^{z-}, and d^{z-}, with z=1-4) are used to reconstruct the sequence from the left- to the right-hand side while fragments containing the ω termination (w^{z-}, x^{z-}, y^{z-}, and z^{z-}, with z=1-4) allow the sequence to be reconstructed from the right- to the left-hand side. The same colors are used to designate fragment series in the CID spectrum and the top table. These data were recorded at a 1.32 eV collision energy (center-of-mass frame) during 3 min (*i.e.*, 174 scan).

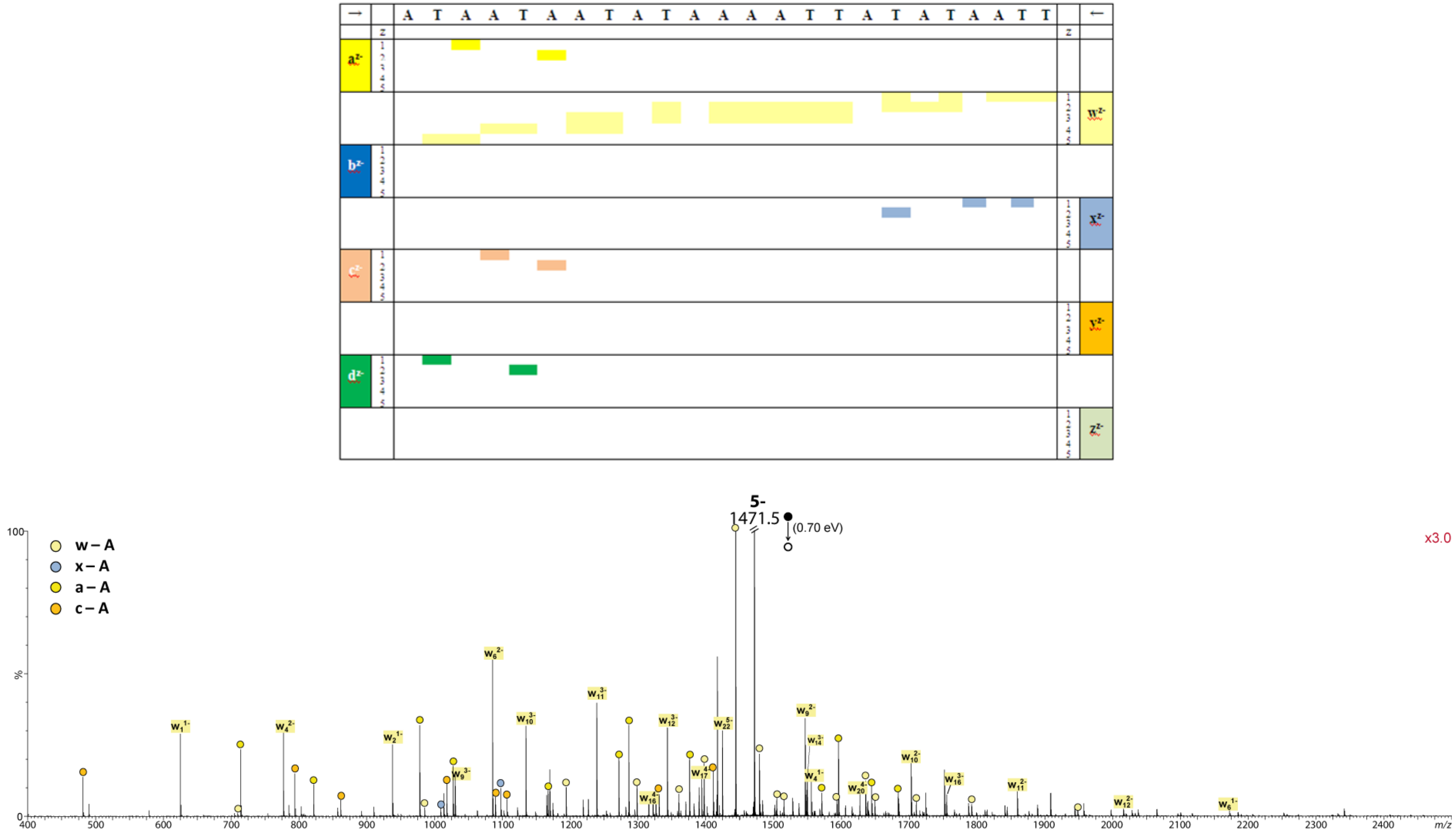

Supplementary Figure 2. ESI-MS/MS of $[M – 5H]^{5-}$ at *m/z* 1471.5 for the digitally-encoded single-strand DNA containing 24 bits (3 bytes) of information coding for ICS (Supplementary Table 1, Entry 2). This simplified model was constructed using only adenine and thymine nucleotides and is therefore not illustrative of ATGC-based codes that are generally used in DNA data storage. This spectrum mainly exhibits w-type ions, allowing here a nearly complete sequence coverage (top table), as well as numerous secondary products formed after primary fragments have experienced one (as annotated by circles) or multiple (not annotated for the sake of clarity) losses of A bases, as commonly reported during CID of oligonucleotides. The same colors are used to designate fragment series in the CID spectrum and the top table. These data were recorded at a 0.70 eV collision energy (center-of-mass frame) during 3 min (*i.e.*, 174 scan).

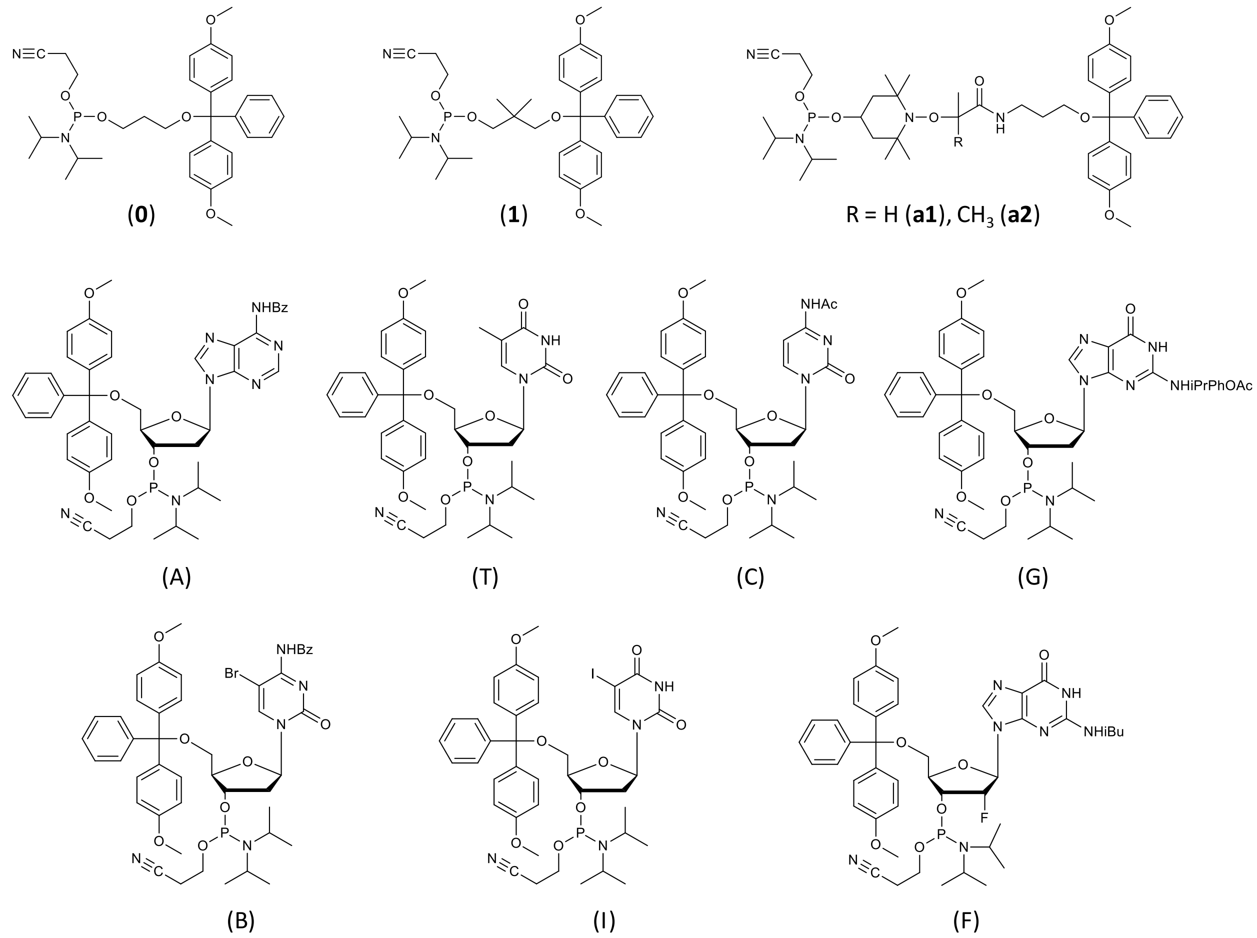

Supplementary Figure 3. Molecular structures of the phosphoramidite monomers used in the present work.

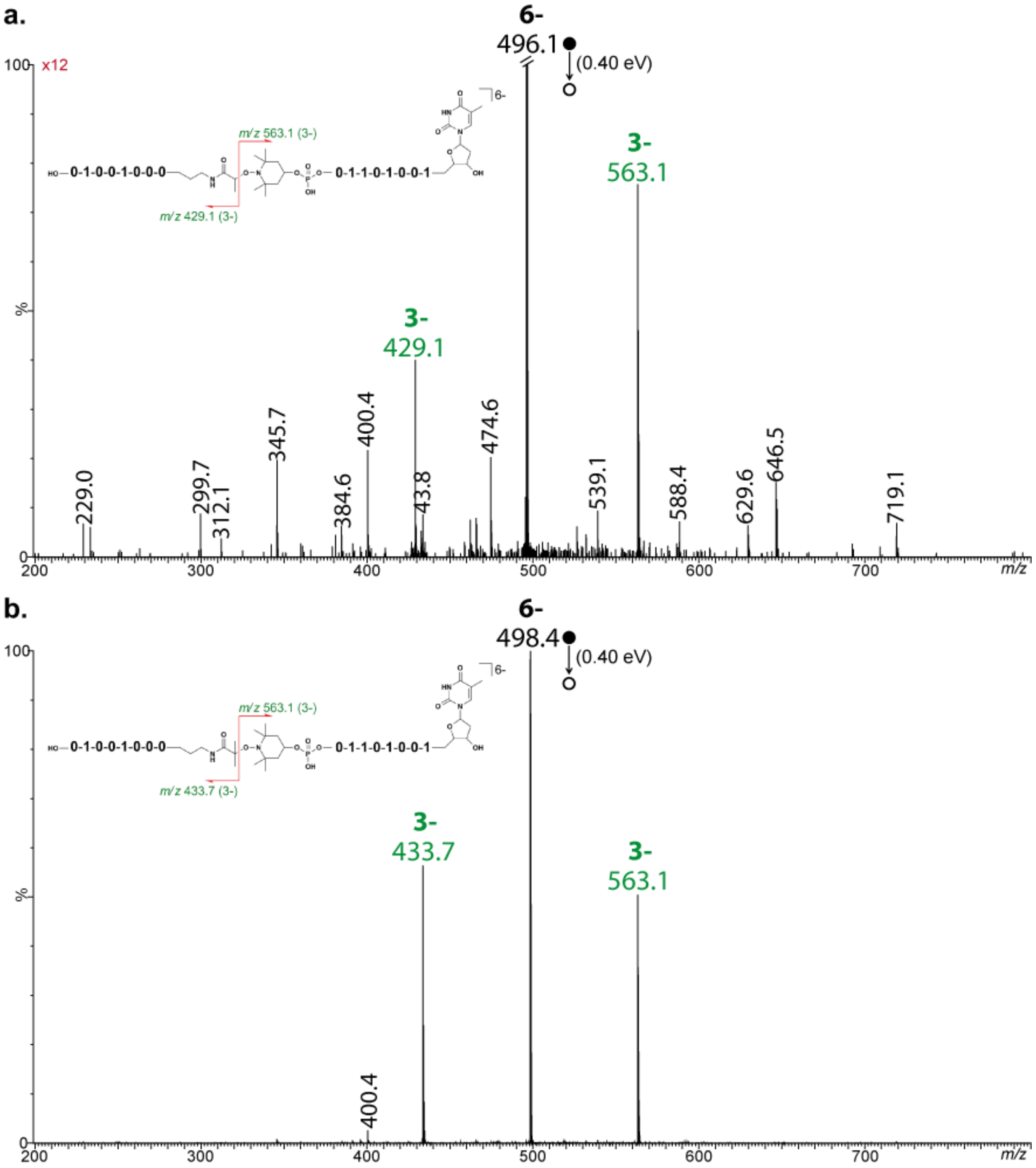

Supplementary Figure 4. ESI-MS/MS of $[M - 6H]^{6-}$ for a 2-byte poly(phosphodiester) with the inter-byte spacer containing (**a**) the monomethylated alkoxyamine **a1** (Supplementary Table 1, Entry 3) or (**b**) the dimethylated alkoxyamine **a2** (Supplementary Table 1, Entry 4). Both spectra were recorded at the same 0.40 eV collision energy (center-of-mass frame) during 0.4 min (*i.e.*, 22 scan). Imparted activation energy is shown to readily allow homolysis of the C–ON bond in the di-methylated alkoxyamine **a2** of *m/z* 498.4 (**b**), as revealed by the main production of the two expected product ions (annotated in green). In contrast, at this energy level, dissociation of phosphate linkages within each byte competes with the C–ON bond homolysis in the mono-methylated alkoxyamine **a1** of *m/z* 496.1 (**a**), leading to production of numerous inner-byte fragments (annotated in black) in addition to the two targeted product ions (annotated in green) that are generated with quite low yield (note that the x12 magnification of this MS/MS spectrum).

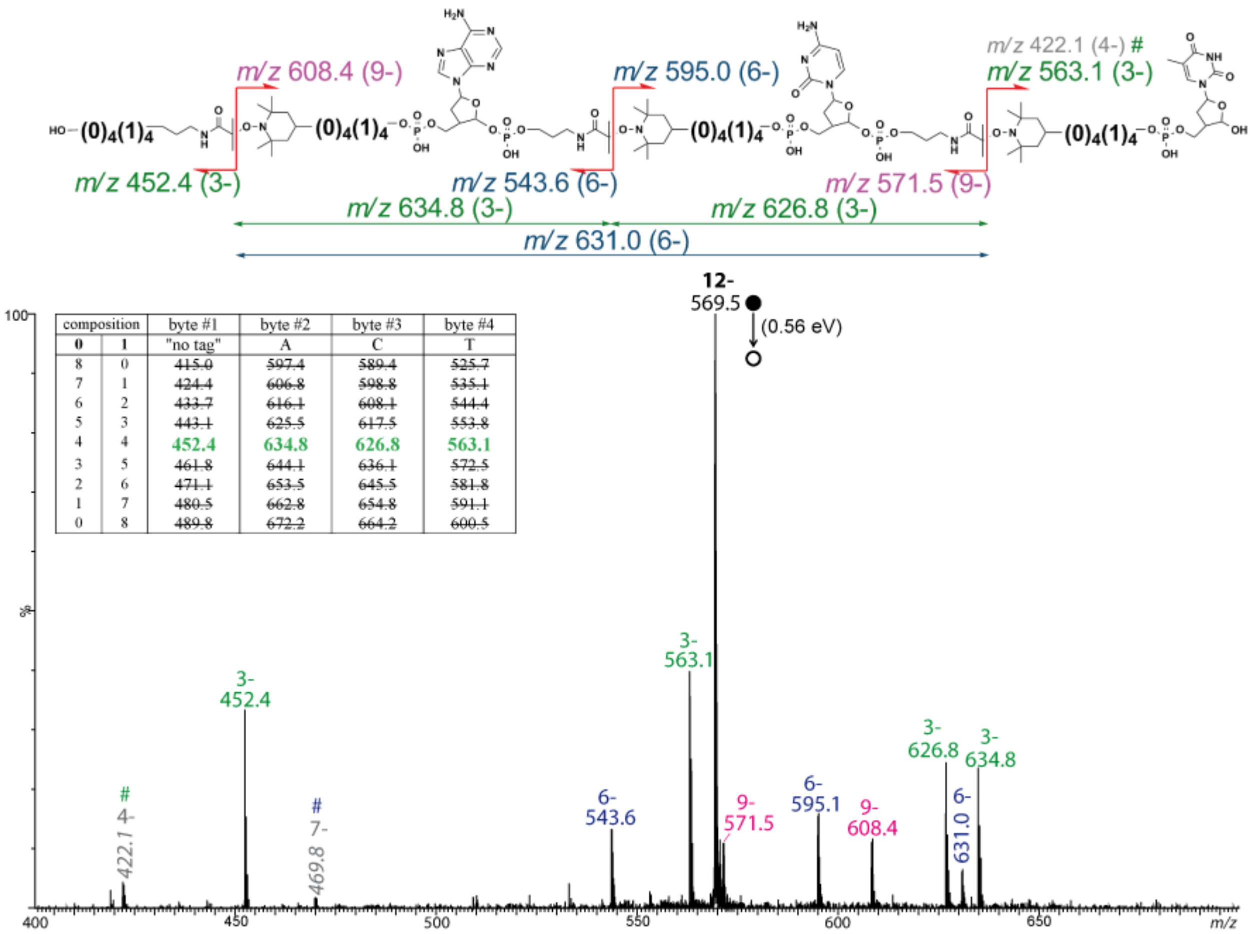

composition		byte #1	byte #2	byte #3	byte #4
**0**	**1**	"no tag"	A	C	T
8	0	~~415.0~~	~~597.4~~	~~589.4~~	~~525.7~~
7	1	~~424.4~~	~~606.8~~	~~598.8~~	~~535.1~~
6	2	~~433.7~~	~~616.1~~	~~608.1~~	~~544.4~~
5	3	~~443.1~~	~~625.5~~	~~617.5~~	~~553.8~~
4	4	**452.4**	**634.8**	**626.8**	**563.1**
3	5	~~461.8~~	~~644.1~~	~~636.1~~	~~572.5~~
2	6	~~471.1~~	~~653.5~~	~~645.5~~	~~581.8~~
1	7	~~480.5~~	~~662.8~~	~~654.8~~	~~591.1~~
0	8	~~489.8~~	~~672.2~~	~~664.2~~	~~600.5~~

Supplementary Figure 5. ESI-MS/MS of $[M - 12H]^{12-}$ (*m/z* 569.5) for the 4-byte poly(phosphodiester) containing 4 times the same **01110100** byte (Supplementary Table 1, Entry 6). Although all bytes exhibit the same **0/1** composition (top structure), they are released as triply deprotonated species at distinct *m/z* values as they carry a different byte-tag as a function of their location in the polymer chain (see inset table). These data were recorded at a 0.56 eV collision energy (center-of-mass frame) during 1 min (*i.e.*, 57 scan).

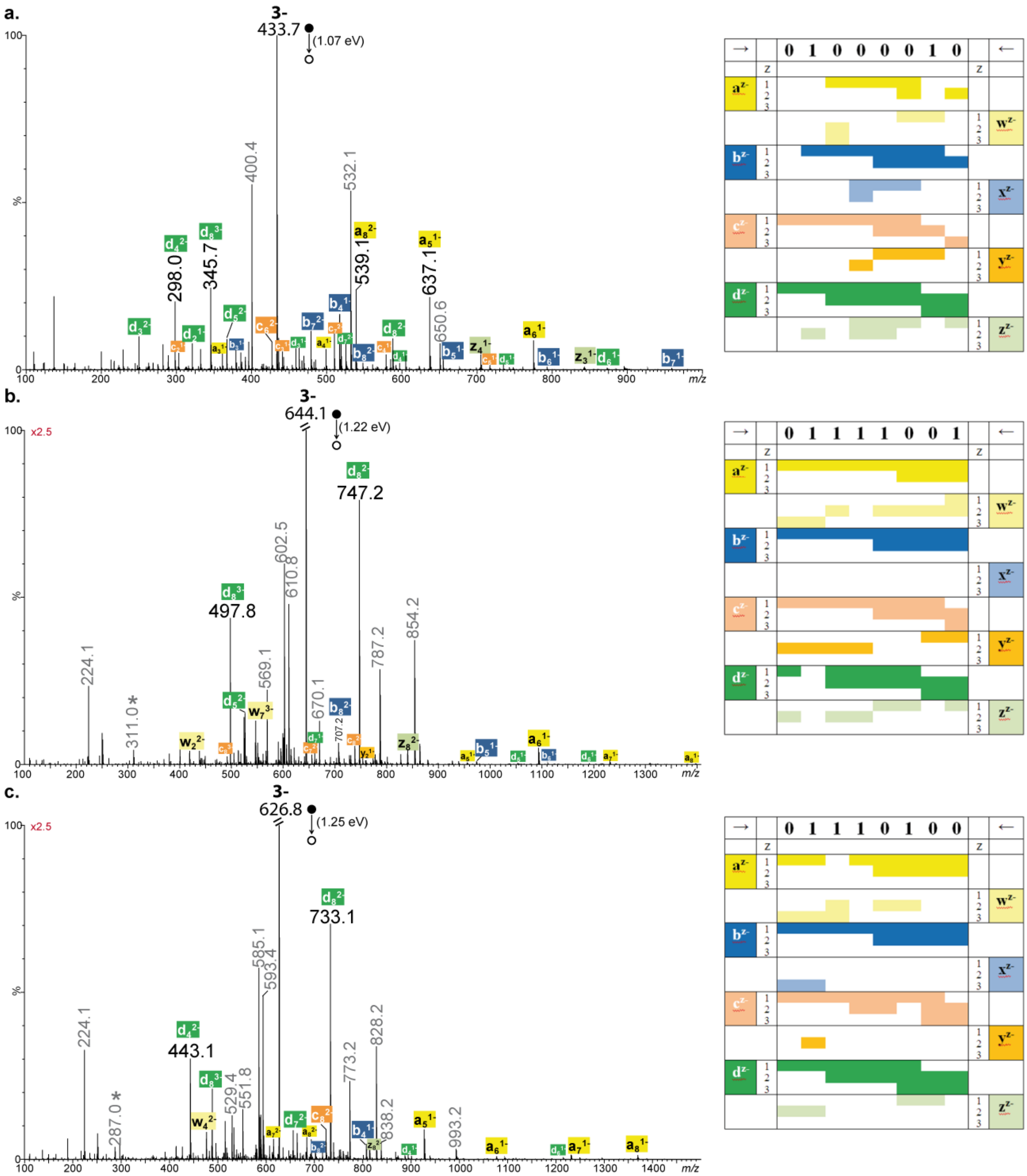

Supplementary Figure 6. Sequencing of triply deprotonated byte-fragments released from the $[M - 12H]^{12-}$ precursor ion (*m/z* 567.0) of the 4-byte poly(phosphodiester) coding for Byte (Supplementary Table 1, Entry 5). Pseudo-MS^3 spectra (left) and associated sequence coverage (right) for (**a**) the *m/z* 433.7 fragment containing a $(\mathbf{0})_6(\mathbf{1})_2$ byte holding no tag, consistent with the expected **01000010** sequence of the 1st byte, (**b**) the *m/z* 644.1 fragment containing a $(\mathbf{0})_3(\mathbf{1})_5$ byte holding tag A, consistent with the expected **01111001** sequence of the 2nd byte, and (**c**) the *m/z* 626.8 fragment containing a $(\mathbf{0})_4(\mathbf{1})_4$ byte holding tag C, consistent with the expected **01110100** sequence of the 3rd byte. Peaks annotated in grey correspond to products formed during reactions induced by the carbon-centered radical, with those designated by an asterisk being diagnostic of the tagging base (see Supplementary Figure 39). These data were recorded during 3 min (*i.e.*, 174 scan) using collision energies as indicated in the center-of-mass frame.

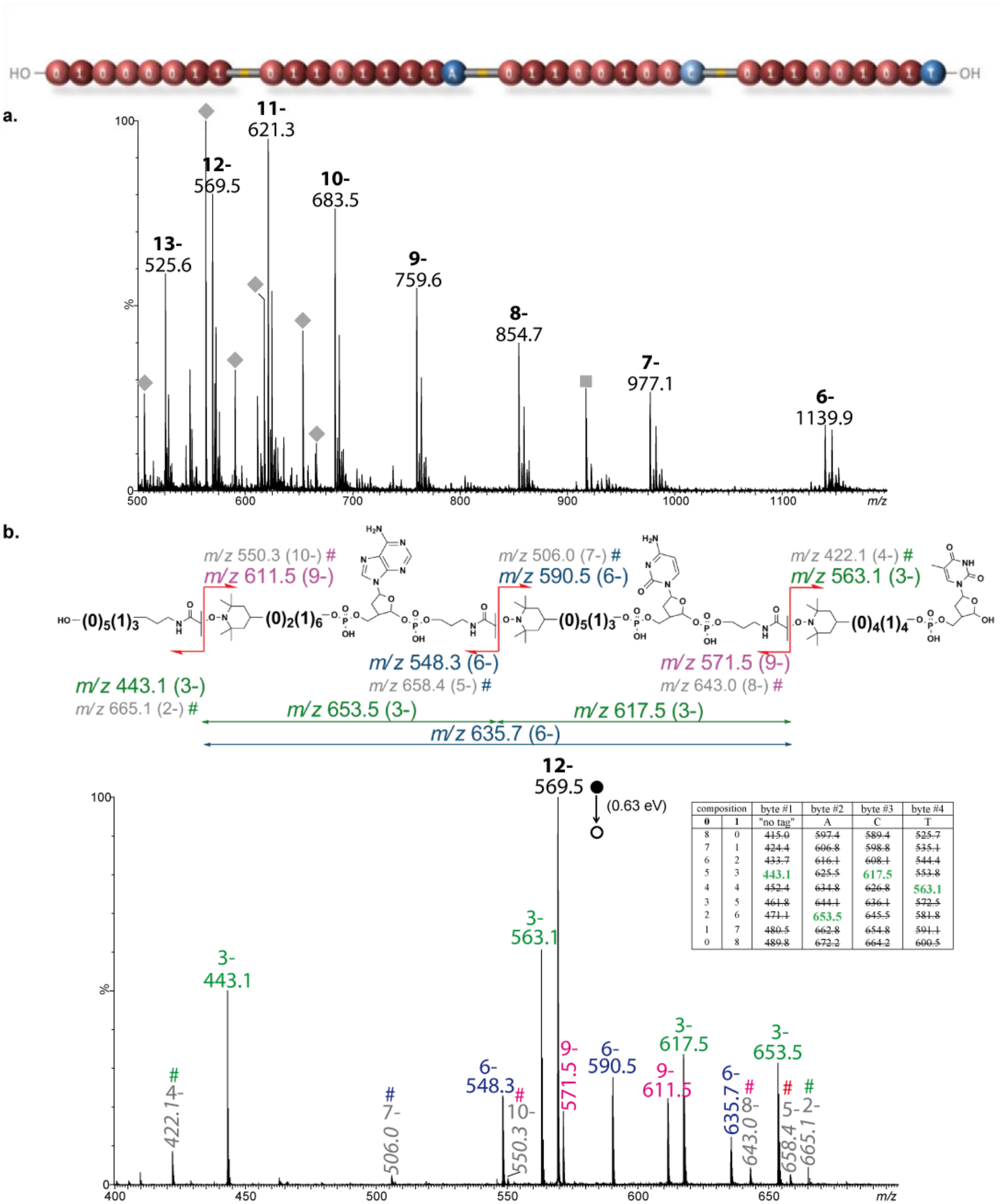

composition		byte #1	byte #2	byte #3	byte #4
**0**	**1**	"no tag"	A	C	T
8	0	~~415.0~~	~~597.4~~	~~589.4~~	~~525.7~~
7	1	~~424.4~~	~~606.8~~	~~598.8~~	~~535.1~~
6	2	~~433.7~~	~~616.1~~	~~608.1~~	~~544.4~~
5	3	**443.1**	~~625.5~~	**617.5**	~~553.8~~
4	4	~~452.4~~	~~634.8~~	~~626.8~~	**563.1**
3	5	~~461.8~~	~~644.1~~	~~636.1~~	~~572.5~~
2	6	~~471.1~~	**653.5**	~~645.5~~	~~581.8~~
1	7	~~480.5~~	~~662.8~~	~~654.8~~	~~591.1~~
0	8	~~489.8~~	~~672.2~~	~~664.2~~	~~600.5~~

Supplementary Figure 7. Sequencing of a 4-bytes polymer that contains the ASCII-encoded word Code (Supplementary Table 1, Entry 7). (**a**) Negative ion mode ESI mass spectrum (MS^1). The bold numbers represent the different charge states observed for the polymer. Grey diamonds and grey squares indicate in-source fragments and synthesis impurities, respectively. (**b**) ESI-MS^2 spectrum of the $[M\text{-}12H]^{12-}$ precursor ion at *m/z* 569.5, where *m/z* values measured for triply charged byte-fragments (in green) reveal both their **0**/**1** composition and their initial location in the polymeric chain (see inset table). Other fragment assignment is indicated in top dissociation scheme. These data were recorded at a 0.63 eV collision energy (center-of-mass frame) during 1 min (*i.e.*, 57 scan).

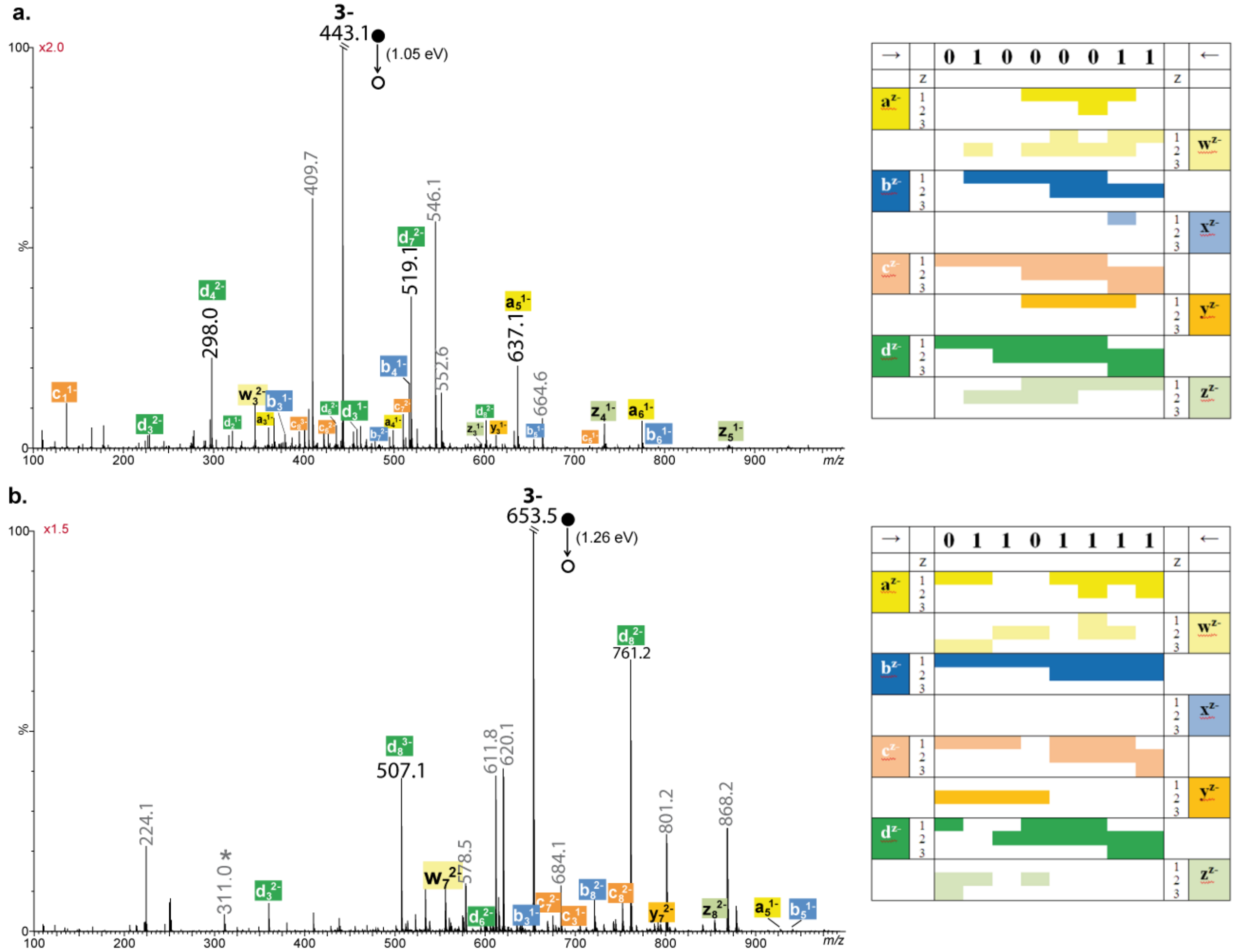

Supplementary Figure 8. Sequencing of a 4-bytes polymer that contains the ASCII-encoded word Code (Supplementary Table 1, Entry 7). Pseudo-MS^3 spectra (left) and associated sequence coverage (right) for (**a**) the *m/z* 443.1 fragment containing a (**0**)$_5$(**1**)$_3$ byte holding no tag, consistent with the expected **01000011** sequence of the 1st byte, and (**b**) the *m/z* 653.5 fragment containing a (**0**)$_2$(**1**)$_6$ byte holding tag A, consistent with the expected **01101111** sequence of the 2nd byte. Peaks annotated in grey correspond to products formed during reactions induced by the carbon-centered radical, with those designated by an asterisk being diagnostic of the tagging base (see Supplementary Figure 39). These data were recorded during 3 min (*i.e.*, 174 scan) using collision energies as indicated in the center-of-mass frame.

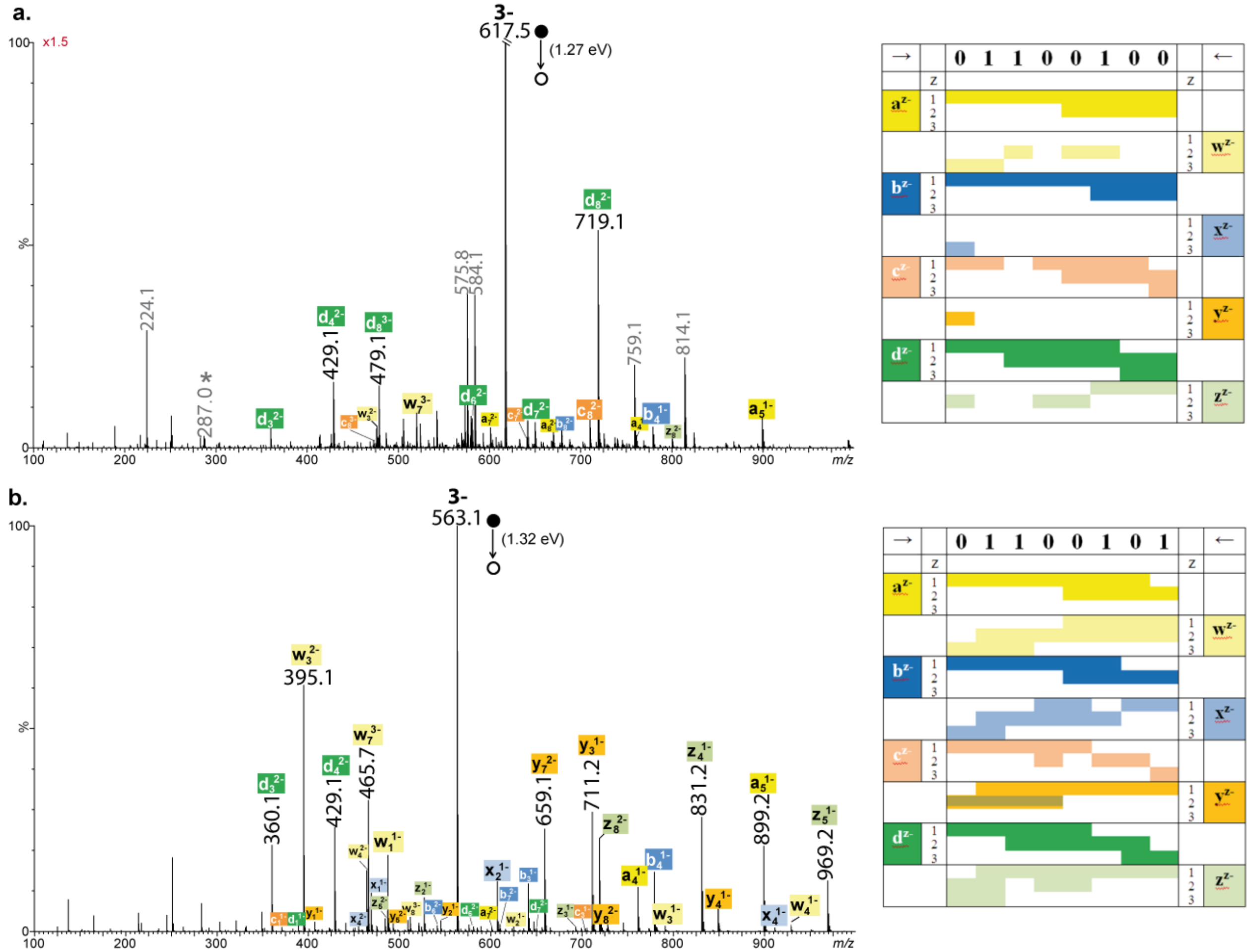

Supplementary Figure 9. Sequencing of a 4-bytes polymer that contains the ASCII-encoded word Code (Supplementary Table 1, Entry 7). Pseudo-MS^3 spectra (left) and associated sequence coverage (right) for (**a**) the *m/z* 617.5 fragment containing a $(\mathbf{0})_5(\mathbf{1})_3$ byte holding tag C, consistent with the expected **01100100** sequence of the 3rd byte, and (**b**) the *m/z* 563.1 fragment containing a $(\mathbf{0})_4(\mathbf{1})_4$ byte holding tag T, consistent with the expected **01100101** sequence of the 4th byte. Peaks annotated in grey correspond to products formed during reactions induced by the carbon-centered radical, with those designated by an asterisk being diagnostic of the tagging base (see Supplementary Figure 39). These data were recorded during 3 min (*i.e.*, 174 scan) using collision energies as indicated in the center-of-mass frame.

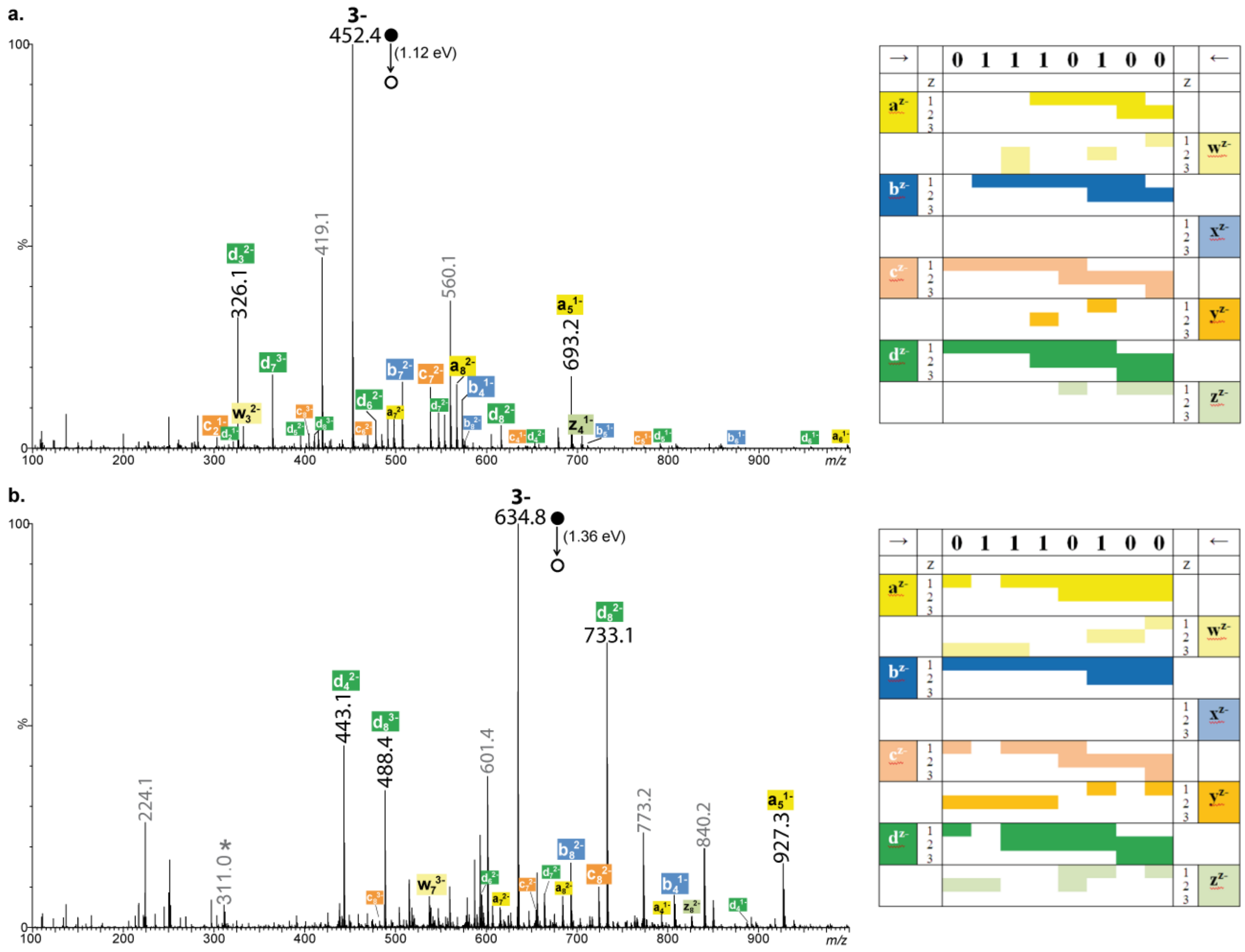

Supplementary Figure 10. Sequencing of triply deprotonated byte-fragments released from the [M – 12H]$^{12-}$ precursor ion (*m/z* 569.5) of the 4-byte poly(phosphodiester) containing 4 times the same **01110100** byte (Supplementary Table 1, Entry 6). Pseudo-MS^3 spectra (left) and associated sequence coverage (right) for (**a**) the *m/z* 452.4 fragment containing a (**0**)$_4$(**1**)$_4$ byte holding no tag, consistent with the expected **01110100** sequence of the 1st byte, and (**b**) the *m/z* 635.1 fragment containing a (**0**)$_4$(**1**)$_4$ byte holding tag A, consistent with the expected **01110100** sequence of the 2nd byte. Peaks annotated in grey correspond to products formed during reactions induced by the carbon-centered radical, with those designated by an asterisk being diagnostic of the tagging base (see Supplementary Figure 39). These data were recorded during 3 min (*i.e.*, 174 scan) using collision energies as indicated in the center-of-mass frame.

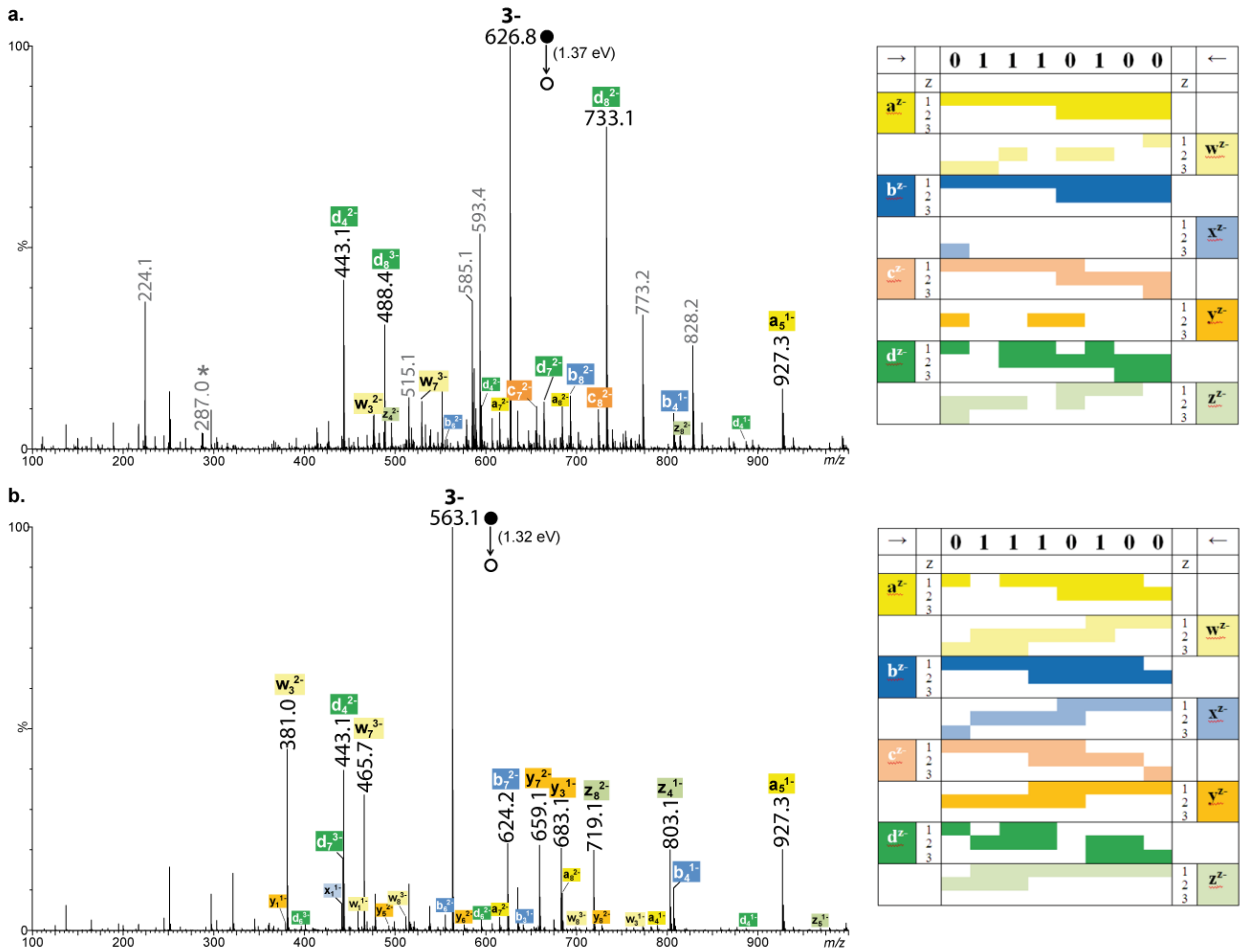

Supplementary Figure 11. Sequencing of triply deprotonated byte-fragments released from the $[M - 12H]^{12-}$ precursor ion (*m/z* 569.5) of the 4-byte poly(phosphodiester) containing 4 times the same **01110100** byte (Supplementary Table 1, Entry 6). Pseudo-MS^3 spectra (left) and associated sequence coverage (right) for (**a**) the *m/z* 626.8 fragment containing a $(\mathbf{0})_4(\mathbf{1})_4$ byte holding tag C, consistent with the expected **01110100** sequence of the 3rd byte, and (**b**) the *m/z* 563.1 fragment containing a $(\mathbf{0})_4(\mathbf{1})_4$ byte holding tag T, consistent with the expected **01110100** sequence of the 4th byte. Peaks annotated in grey correspond to products formed during reactions induced by the carbon-centered radical, with those designated by an asterisk being diagnostic of the tagging base (see Supplementary Figure 39). These data were recorded during 3 min (*i.e.*, 174 scan) using collision energies as indicated in the center-of-mass frame.

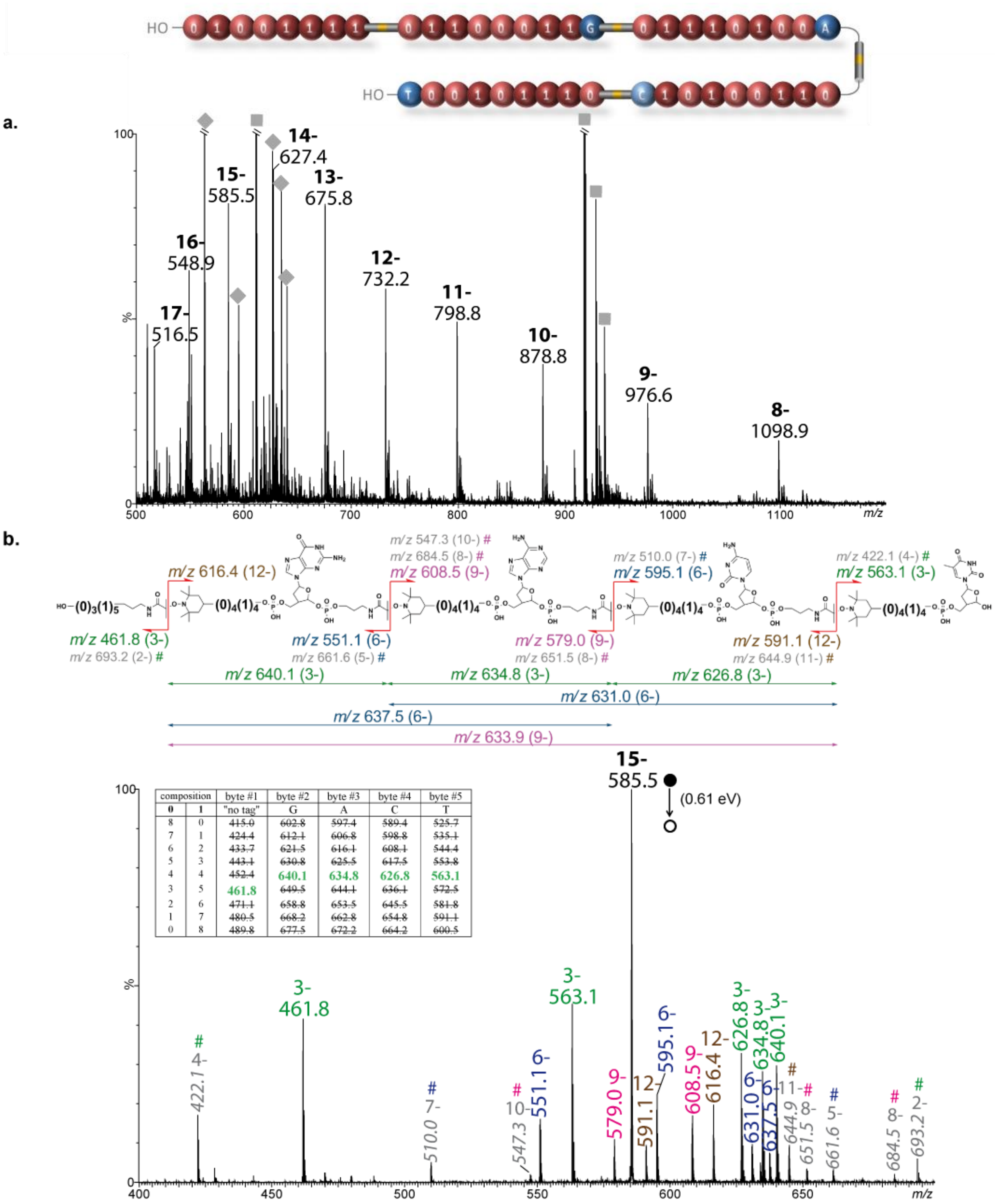

composition		byte #1	byte #2	byte #3	byte #4	byte #5
**0**	**1**	"no tag"	G	A	C	T
8	0	~~415.0~~	~~602.8~~	~~597.4~~	~~589.4~~	~~525.7~~
7	1	~~424.4~~	~~612.1~~	~~606.8~~	~~598.8~~	~~535.1~~
6	2	~~433.7~~	~~621.5~~	~~616.1~~	~~608.1~~	~~544.4~~
5	3	~~443.1~~	~~630.8~~	~~625.5~~	~~617.5~~	~~553.8~~
4	4	~~452.4~~	**640.1**	**634.8**	**626.8**	**563.1**
3	5	**461.8**	~~649.5~~	~~644.1~~	~~636.1~~	~~572.5~~
2	6	~~471.1~~	~~658.8~~	~~653.5~~	~~645.5~~	~~581.8~~
1	7	~~480.5~~	~~668.2~~	~~662.8~~	~~654.8~~	~~591.1~~
0	8	~~489.8~~	~~677.5~~	~~672.2~~	~~664.2~~	~~600.5~~

Supplementary Figure 12. Sequencing of a 5-bytes polymer that contains the ASCII-encoded word Octet (Supplementary Table 1, Entry 8). (**a**) Negative ion mode ESI mass spectrum (MS^1). Bold numbers represent the different charge states observed for the polymer. Grey diamonds and grey squares indicate in-source fragments and synthesis impurities, respectively. (**b**) ESI-MS^2 spectrum of the $[M\text{-}15H]^{15-}$ precursor ion at *m/z* 585.5, where *m/z* values measured for triply charged byte-fragments (in green) reveal both their **0**/**1** composition and their initial location in the polymeric chain (see inset table). Other fragment assignment is indicated in top dissociation scheme. These data were recorded at a 0.61 eV collision energy (center-of-mass frame) during 1 min (*i.e.*, 57 scan).

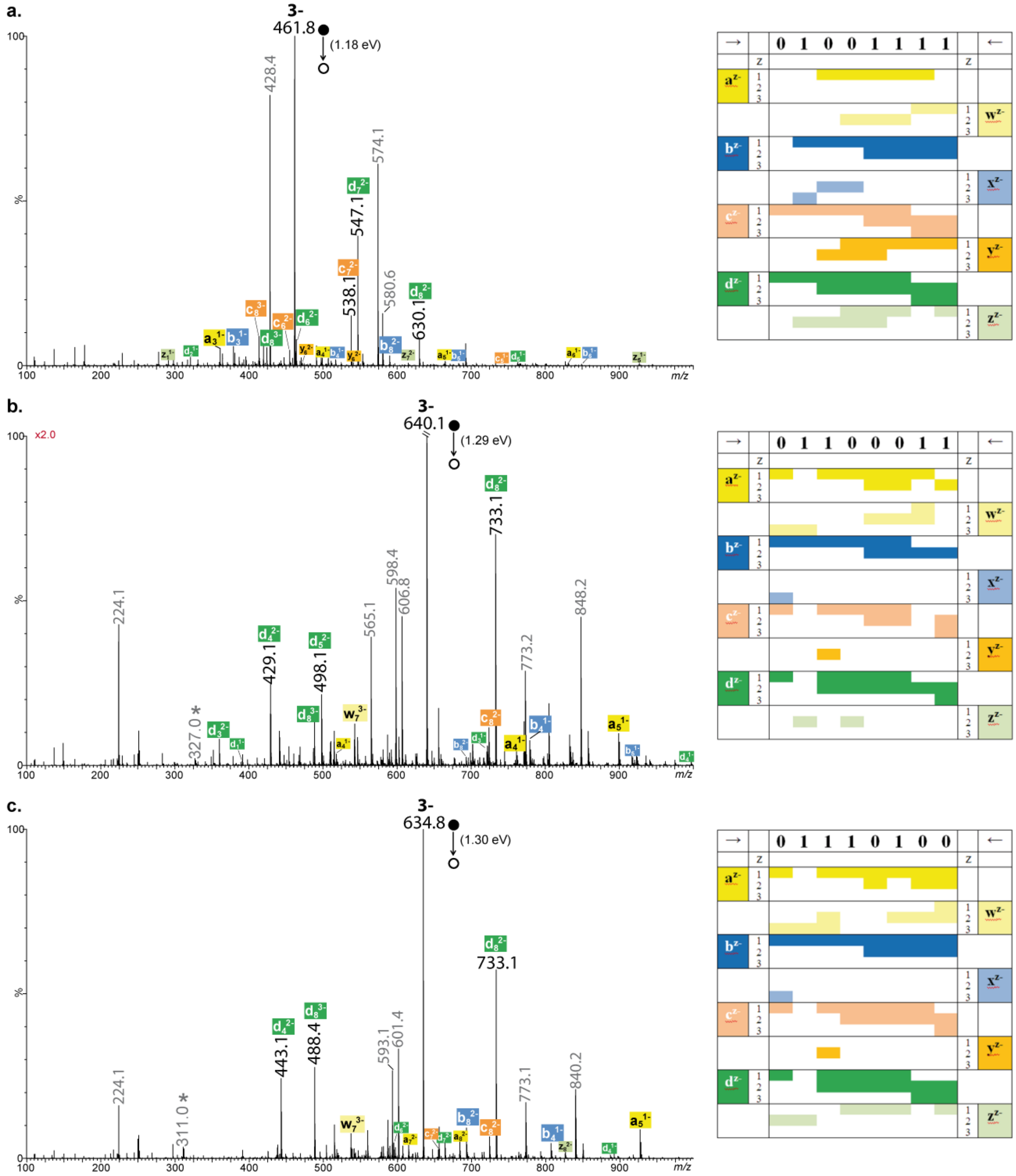

Supplementary Figure 13. Sequencing of a 5-bytes polymer that contains the ASCII-encoded word Octet (Supplementary Table 1, Entry 8). Pseudo-MS^3 spectra (left) and associated sequence coverage (right) for (**a**) the *m/z* 461.8 fragment containing a (**0**)$_3$(**1**)$_5$ byte holding no tag, consistent with the expected **01001111** sequence of the 1st byte, (**b**) the *m/z* 640.1 fragment containing a (**0**)$_4$(**1**)$_4$ byte holding tag G, consistent with the expected **01100011** sequence of the 2nd byte, and (**c**) the *m/z* 634.8 fragment containing a (**0**)$_4$(**1**)$_4$ byte holding tag A, consistent with the expected **01110100** sequence of the 3rd byte. Peaks annotated in grey correspond to products formed during reactions induced by the carbon-centered radical, with those designated by an asterisk being diagnostic of the tagging base (see Supplementary Figure 39). These data were recorded during 3 min (*i.e.*, 174 scan) using collision energies as indicated in the center-of-mass frame.

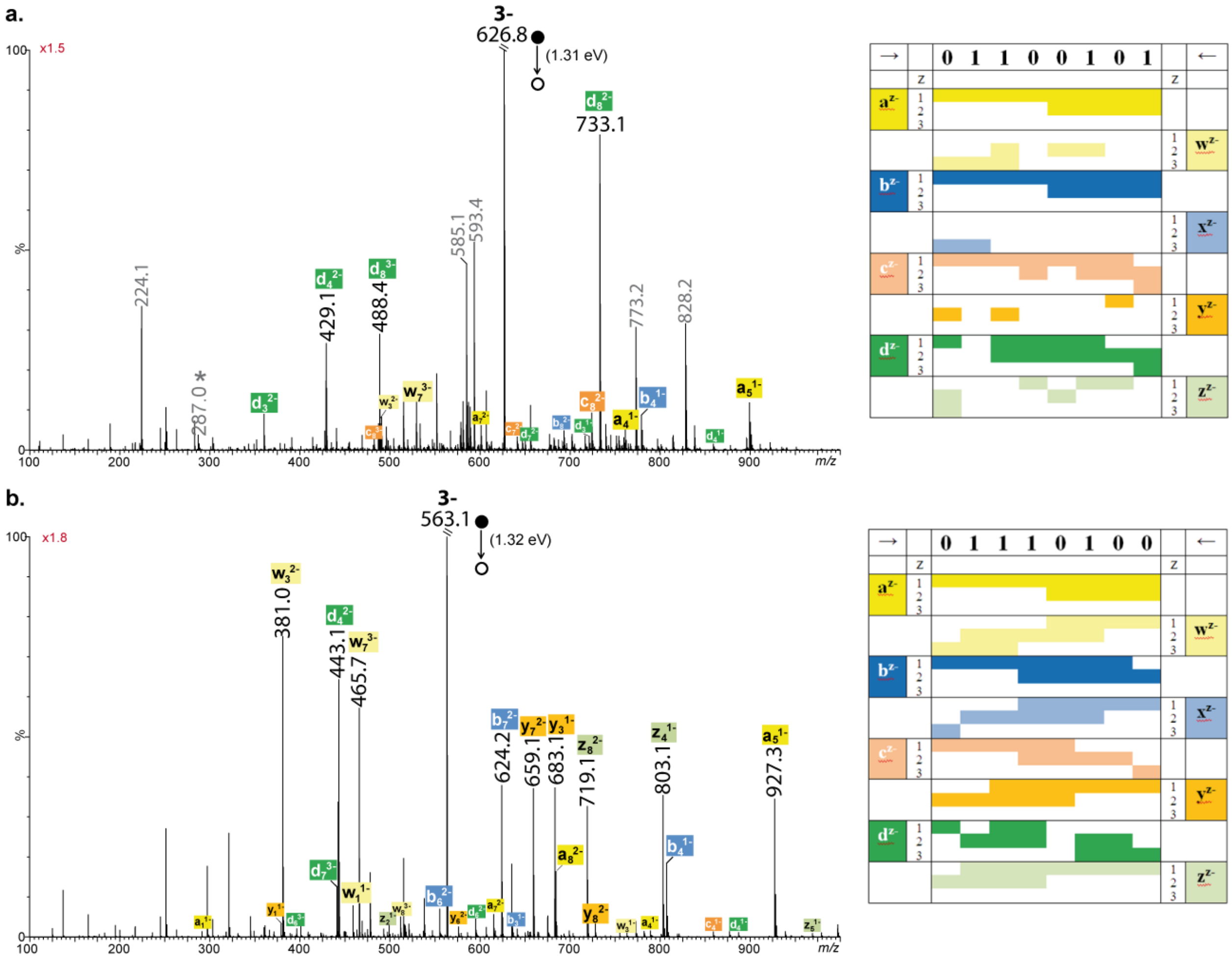

Supplementary Figure 14. Sequencing of a 5-bytes polymer that contains the ASCII-encoded word Octet (Supplementary Table 1, Entry 8). Pseudo-MS^3 spectra (left) and associated sequence coverage (right) for (**a**) the *m/z* 626.8 fragment containing a $(\mathbf{0})_4(\mathbf{1})_4$ byte holding tag C, consistent with the expected **01100101** sequence of the 4th byte, and (**b**) the *m/z* 563.1 fragment containing a $(\mathbf{0})_4(\mathbf{1})_4$ byte holding tag T, consistent with the expected **01110100** sequence of the 5th byte. Peaks annotated in grey correspond to products formed during reactions induced by the carbon-centered radical, with those designated by an asterisk being diagnostic of the tagging base (see Supplementary Figure 39). These data were recorded during 3 min (*i.e.*, 174 scan) using collision energies as indicated in the center-of-mass frame.

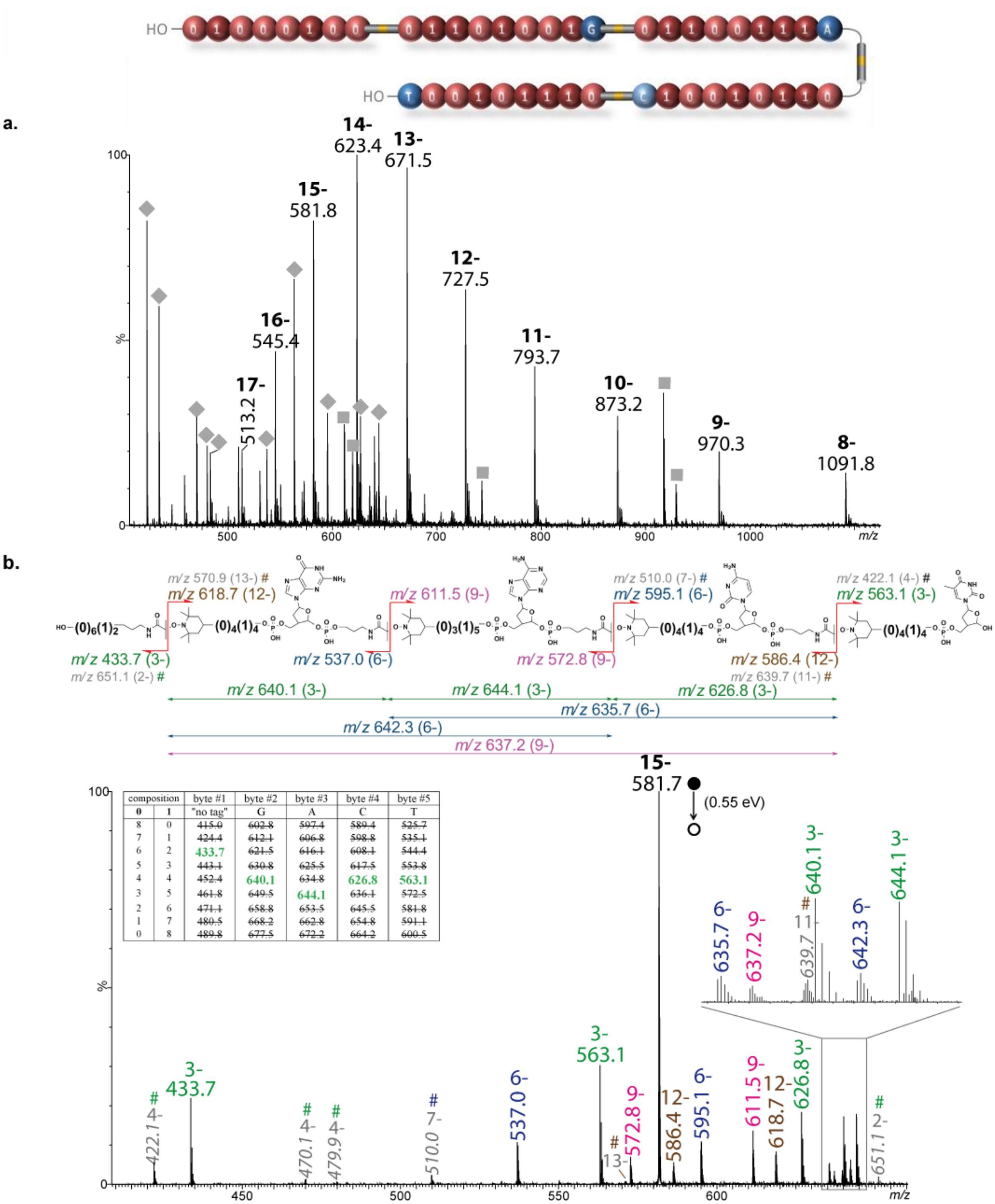

composition		byte #1	byte #2	byte #3	byte #4	byte #5
**0**	**1**	"no tag"	G	A	C	T
8	0	~~415.0~~	~~602.8~~	~~597.4~~	~~589.4~~	~~525.7~~
7	1	~~424.4~~	~~612.1~~	~~606.8~~	~~598.8~~	~~535.1~~
6	2	**433.7**	~~621.5~~	~~616.1~~	~~608.1~~	~~544.4~~
5	3	~~443.1~~	~~630.8~~	~~625.5~~	~~617.5~~	~~553.8~~
4	4	~~452.4~~	**640.1**	~~634.8~~	**626.8**	**563.1**
3	5	~~461.8~~	~~649.5~~	**644.1**	~~636.1~~	~~572.5~~
2	6	~~471.1~~	~~658.8~~	~~653.5~~	~~645.5~~	~~581.8~~
1	7	~~480.5~~	~~668.2~~	~~662.8~~	~~654.8~~	~~591.1~~
0	8	~~489.8~~	~~677.5~~	~~672.2~~	~~664.2~~	~~600.5~~

Supplementary Figure 15. Sequencing of a 5-bytes polymer that contains the ASCII-encoded word Digit (Supplementary Table 1, Entry 9). (**a**) Negative ion mode ESI mass spectrum (MS^1). Bold numbers represent the different charge states observed for the polymer. Grey diamonds and grey squares indicate in-source fragments and synthesis impurities, respectively. (**b**) ESI-MS^2 spectrum of the $[M\text{-}15H]^{15-}$ precursor ion at *m/z* 581.7, where *m/z* values measured for triply charged byte-fragments (in green) reveal both their **0**/**1** composition and their initial location in the polymeric chain (see inset table). Other fragment assignment is indicated in top dissociation scheme. These data were recorded at a 0.55 eV collision energy (center-of-mass frame) during 1 min (*i.e.*, 57 scan).

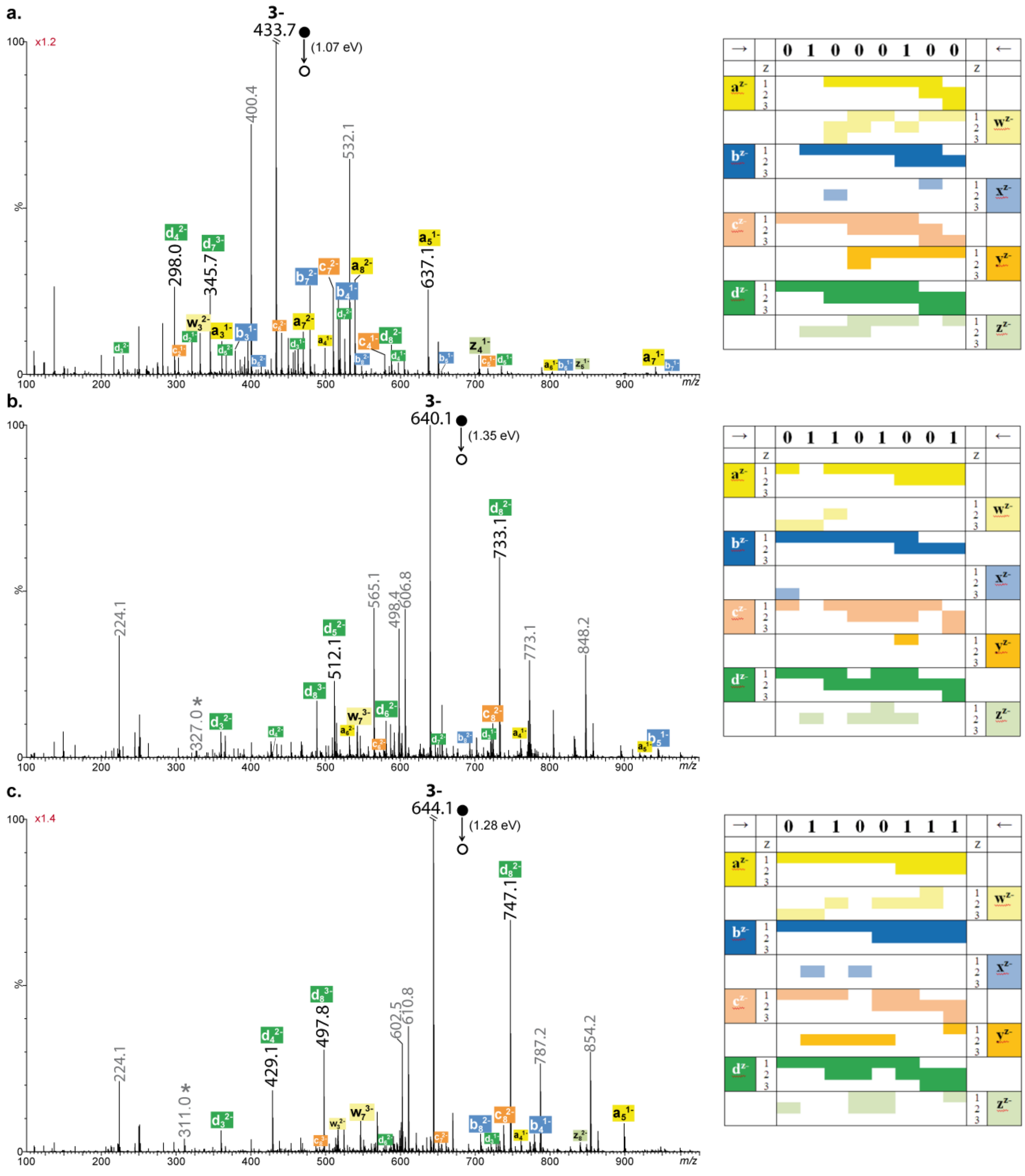

Supplementary Figure 16. Sequencing of a 5-bytes polymer that contains the ASCII-encoded word Digit (Supplementary Table 1, Entry 9). Pseudo-MS^3 spectra (left) and associated sequence coverage (right) for (**a**) the *m/z* 433.7 fragment containing a (**0**)$_6$(**1**)$_2$ byte holding no tag, consistent with the expected **01000100** sequence of the 1st byte, (**b**) the *m/z* 640.1 fragment containing a (**0**)$_4$(**1**)$_4$ byte holding tag G, consistent with the expected **01101001** sequence of the 2nd byte, and (**c**) the *m/z* 644.1 fragment containing a (**0**)$_3$(**1**)$_5$ byte holding tag A, consistent with the expected **01100111** sequence of the 3rd byte. Peaks annotated in grey correspond to products formed during reactions induced by the carbon-centered radical, with those designated by an asterisk being diagnostic of the tagging base (see Supplementary Figure 39). These data were recorded during 3 min (*i.e.*, 174 scan) using collision energies as indicated in the center-of-mass frame.

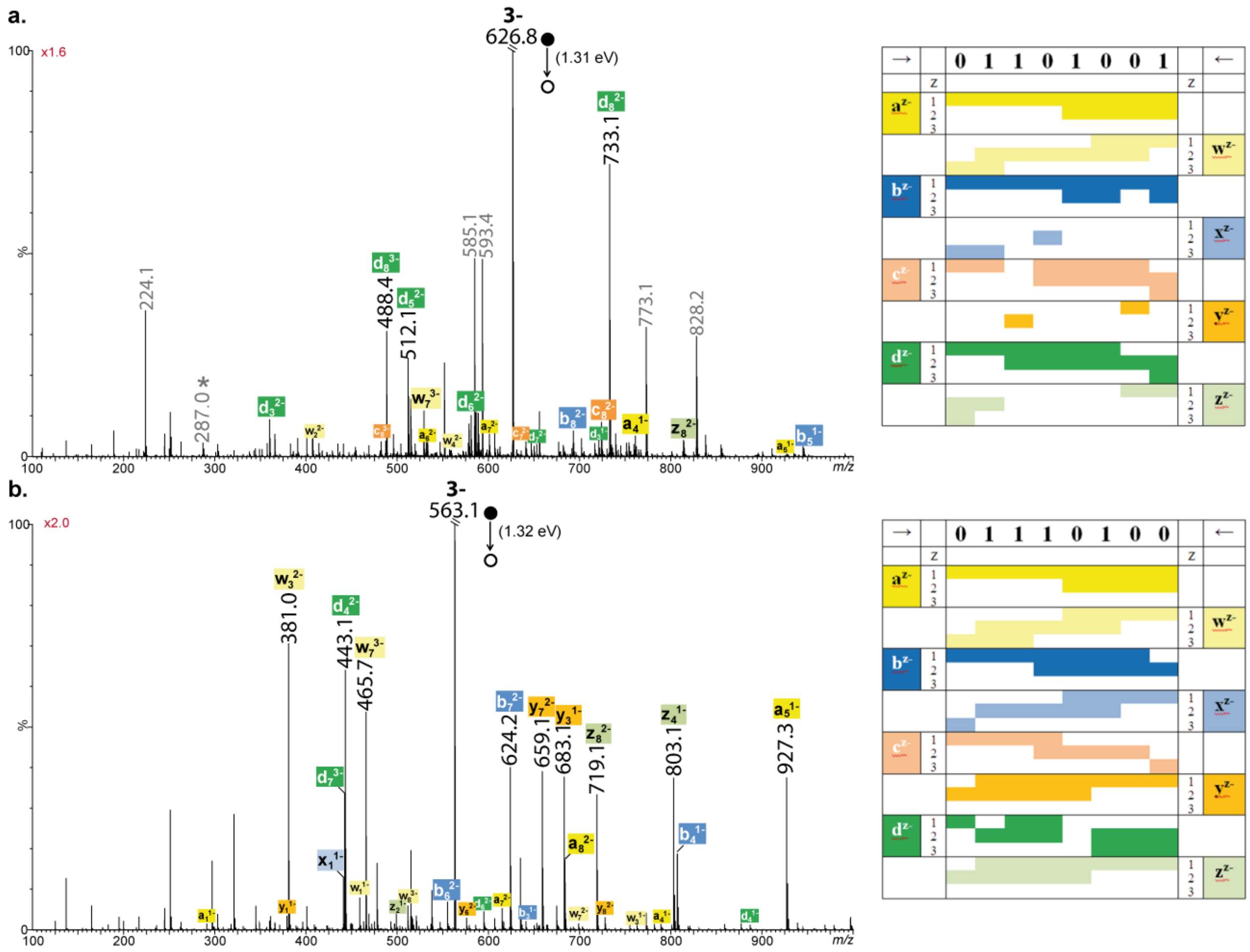

Supplementary Figure 17. Sequencing of a 5-bytes polymer that contains the ASCII-encoded word Digit (Supplementary Table 1, Entry 9). Pseudo-MS^3 spectra (left) and associated sequence coverage (right) for (**a**) the *m/z* 626.8 fragment containing a $(\mathbf{0})_4(\mathbf{1})_4$ byte holding tag C, consistent with the expected **01101001** sequence of the 4th byte, and (**b**) the *m/z* 563.1 fragment containing a $(\mathbf{0})_4(\mathbf{1})_4$ byte holding tag T, consistent with the expected **01110100** sequence of the 5th byte. Peaks annotated in grey correspond to products formed during reactions induced by the carbon-centered radical, with those designated by an asterisk being diagnostic of the tagging base (see Supplementary Figure 39). These data were recorded during 3 min (*i.e.*, 174 scan) using collision energies as indicated in the center-of-mass frame.

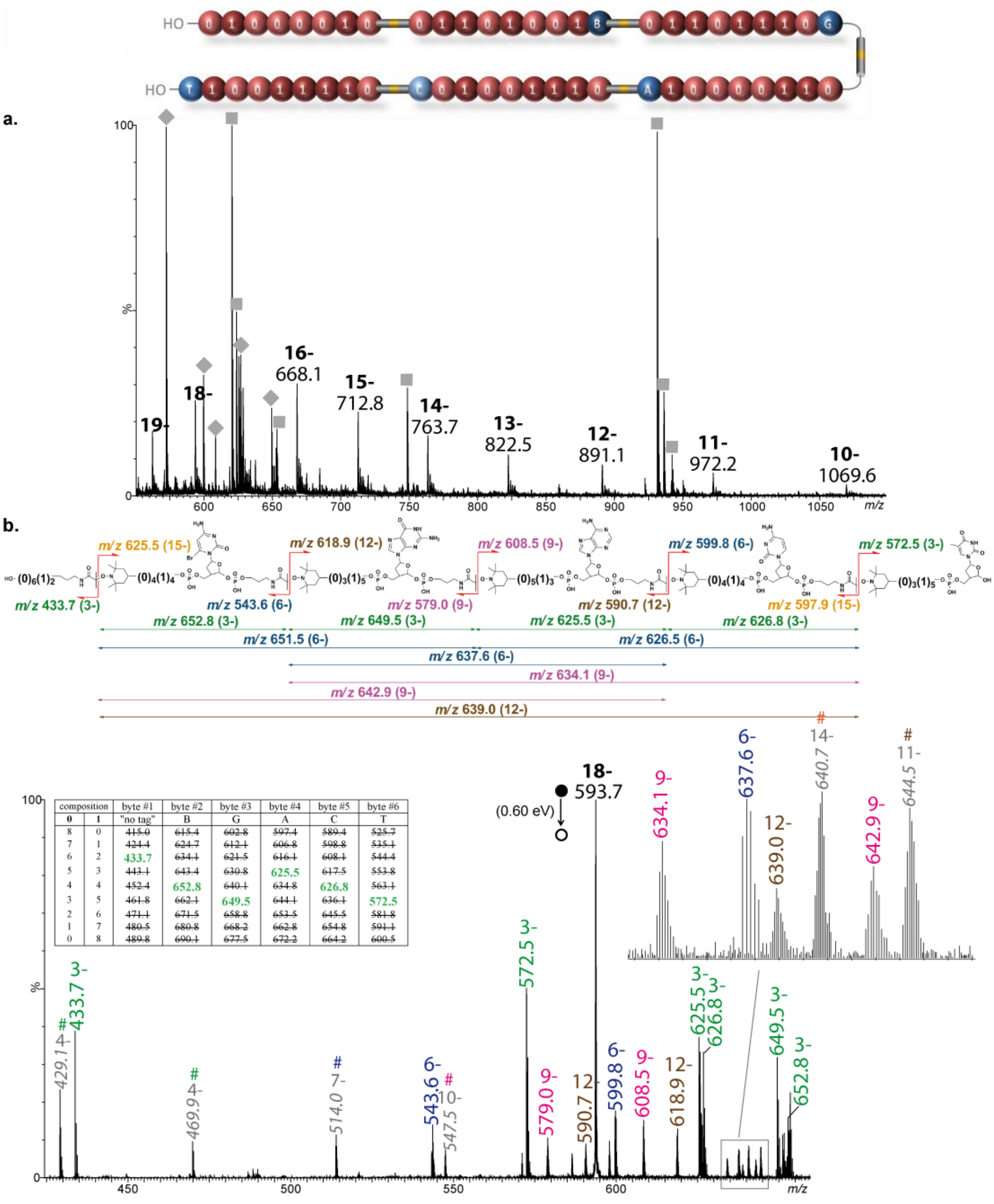

composition		byte #1	byte #2	byte #3	byte #4	byte #5	byte #6
**0**	**1**	"no tag"	B	G	A	C	T
8	0	~~415.0~~	~~615.4~~	~~602.8~~	~~597.4~~	~~589.4~~	~~525.7~~
7	1	~~424.4~~	~~624.7~~	~~612.1~~	~~606.8~~	~~598.8~~	~~535.1~~
6	2	**433.7**	~~634.1~~	~~621.5~~	~~616.1~~	~~608.1~~	~~544.4~~
5	3	~~443.1~~	~~643.4~~	~~630.8~~	**625.5**	~~617.5~~	~~553.8~~
4	4	~~452.4~~	**652.8**	~~640.1~~	~~634.8~~	**626.8**	~~563.1~~
3	5	~~461.8~~	~~662.1~~	**649.5**	~~644.1~~	~~636.1~~	**572.5**
2	6	~~471.1~~	~~671.5~~	~~658.8~~	~~653.5~~	~~645.5~~	~~581.8~~
1	7	~~480.5~~	~~680.8~~	~~668.2~~	~~662.8~~	~~654.8~~	~~591.1~~
0	8	~~489.8~~	~~690.1~~	~~677.5~~	~~672.2~~	~~664.2~~	~~600.5~~

Supplementary Figure 18. Sequencing of a 6-bytes polymer that contains the ASCII-encoded word Binary (Supplementary Table 1, Entry 10). (**a**) Negative ion mode ESI mass spectrum (MS^1). Bold numbers represent the different charge states observed for the polymer. Grey diamonds and grey squares indicate in-source fragments and synthesis impurities, respectively. (**b**) ESI-MS^2 spectrum of the $[M\text{-}18H]^{18-}$ precursor ion at *m/z* 593.7, where *m/z* values measured for triply charged byte-fragments (in green) reveal both their **0**/**1** composition and their initial location in the polymeric chain (see inset table). Other fragment assignment is indicated in top dissociation scheme. These data were recorded at a 0.60 eV collision energy (center-of-mass frame) during 1 min (*i.e.*, 57 scan).

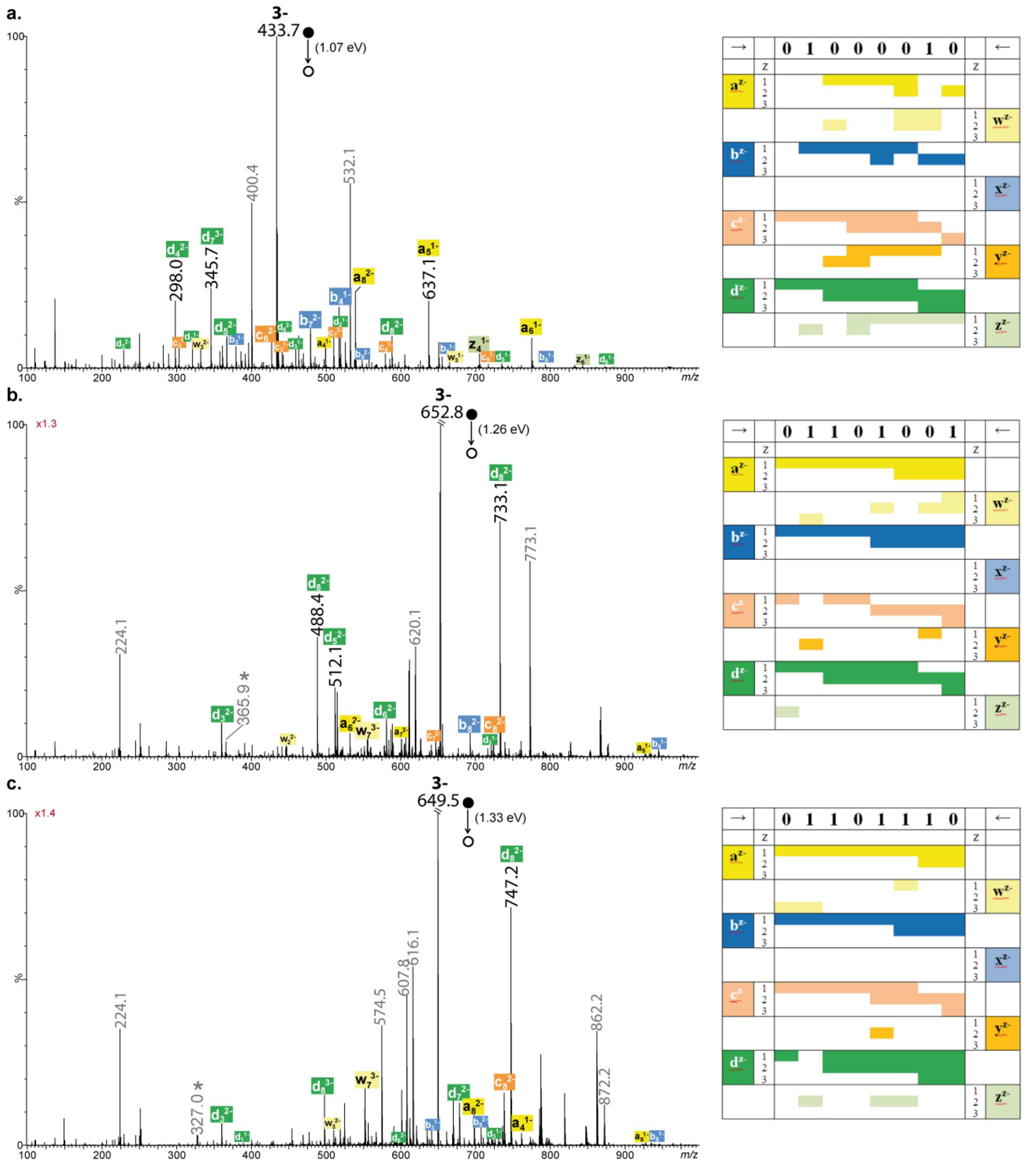

Supplementary Figure 19. Sequencing of a 6-bytes polymer that contains the ASCII-encoded word Binary (Supplementary Table 1, Entry 10). Pseudo-MS^3 spectra (left) and associated sequence coverage (right) for (**a**) the *m/z* 433.7 fragment containing a $(\mathbf{0})_6(\mathbf{1})_2$ byte holding no tag, consistent with the expected **01000010** sequence of the 1st byte, (**b**) the *m/z* 652.8 fragment containing a $(\mathbf{0})_4(\mathbf{1})_4$ byte holding tag B, consistent with the expected **01101001** sequence of the 2nd byte, and (**c**) the *m/z* 649.5 fragment containing a $(\mathbf{0})_3(\mathbf{1})_5$ byte holding tag G, consistent with the expected **01101110** sequence of the 3rd byte. Peaks annotated in grey correspond to products formed during reactions induced by the carbon-centered radical, with those designated by an asterisk being diagnostic of the tagging base (see Supplementary Figure 39). These data were recorded during 3 min (*i.e.*, 174 scan) using collision energies as indicated in the center-of-mass frame.

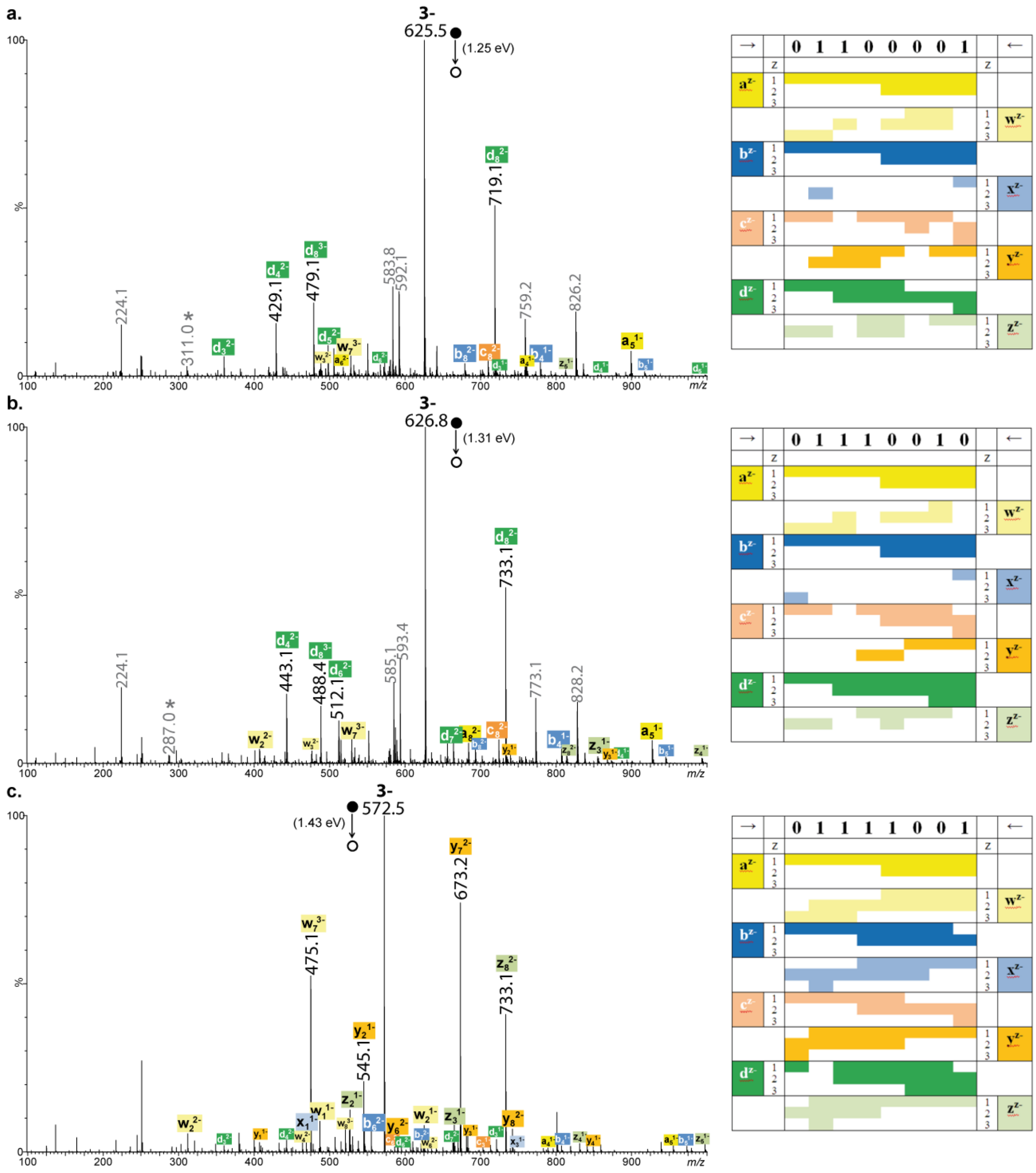

Supplementary Figure 20. Sequencing of a 6-bytes polymer that contains the ASCII-encoded word Binary (Supplementary Table 1, Entry 10). Pseudo-MS^3 spectra (left) and associated sequence coverage (right) for (**a**) the *m/z* 625.5 fragment containing a (**0**)$_5$(**1**)$_3$ byte holding tag A, consistent with the expected **01100001** sequence of the 4th byte, (**b**) the *m/z* 626.8 fragment containing a (**0**)$_4$(**1**)$_4$ byte holding tag C, consistent with the expected **01110010** sequence of the 5th byte, and (**c**) the *m/z* 572.5 fragment containing a (**0**)$_3$(**1**)$_5$ byte holding tag T, consistent with the expected **01111001** sequence of the 6th byte. Peaks annotated in grey correspond to products formed during reactions induced by the carbon-centered radical, with those designated by an asterisk being diagnostic of the tagging base (see Supplementary Figure 39). These data were recorded during 3 min (*i.e.*, 174 scan) using collision energies as indicated in the center-of-mass frame.

a.

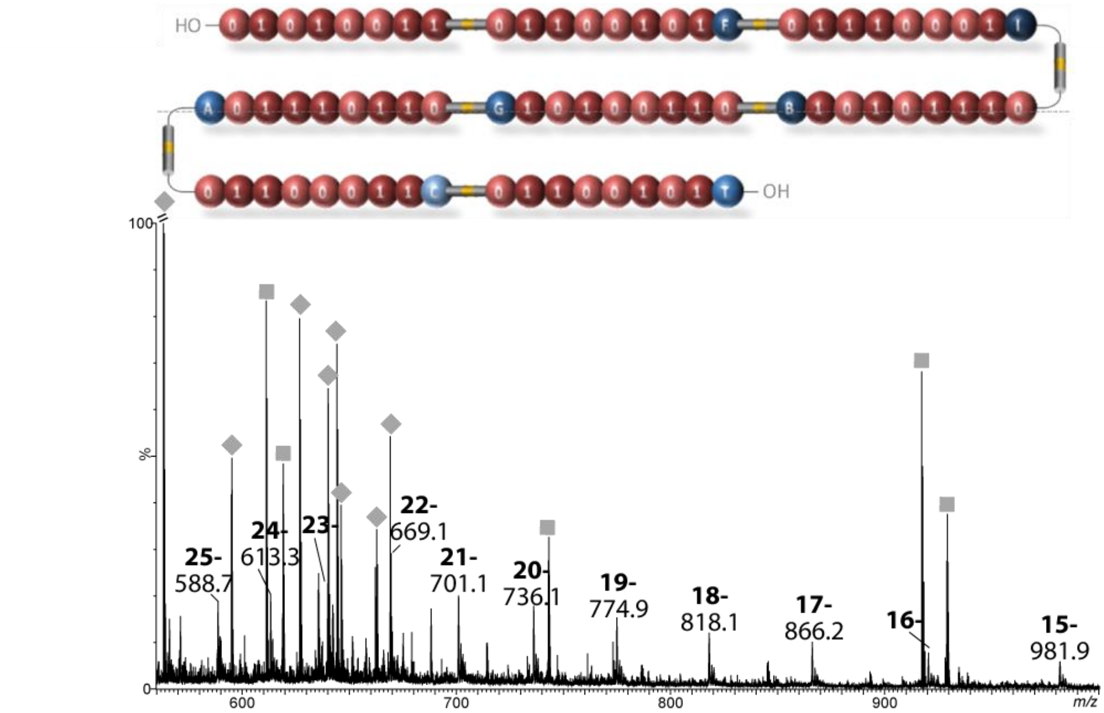

b.

composition		byte #1	byte #2	byte #3	byte #4	byte #5	byte #6	byte #7	byte #8
0	1	"no tag"	F	I	B	G	A	C	T
8	0	~~415.0~~	~~608.8~~	~~632.7~~	~~615.4~~	~~602.8~~	~~597.4~~	~~589.4~~	~~525.7~~
7	1	~~424.4~~	~~618.1~~	~~641.1~~	~~624.7~~	~~612.1~~	~~606.8~~	~~598.8~~	~~535.1~~
6	2	~~433.7~~	~~627.4~~	~~650.4~~	~~634.1~~	~~621.5~~	~~616.1~~	~~608.1~~	~~544.4~~
5	3	~~443.1~~	~~636.8~~	~~659.8~~	~~643.4~~	~~630.8~~	~~625.5~~	~~617.5~~	~~553.8~~
4	4	**452.4**	**646.1**	**669.1**	~~652.8~~	**640.1**	~~634.8~~	**626.8**	**563.1**
3	5	~~461.8~~	~~655.5~~	~~678.4~~	**662.1**	~~649.5~~	**644.1**	~~636.1~~	~~572.5~~
2	6	~~471.1~~	~~664.8~~	~~687.8~~	~~671.5~~	~~658.8~~	~~653.5~~	~~645.5~~	~~581.8~~
1	7	~~480.5~~	~~674.2~~	~~697.1~~	~~680.8~~	~~668.2~~	~~662.8~~	~~654.8~~	~~591.1~~
0	8	~~489.8~~	~~683.5~~	~~706.5~~	~~690.1~~	~~677.5~~	~~672.2~~	~~664.2~~	~~600.5~~

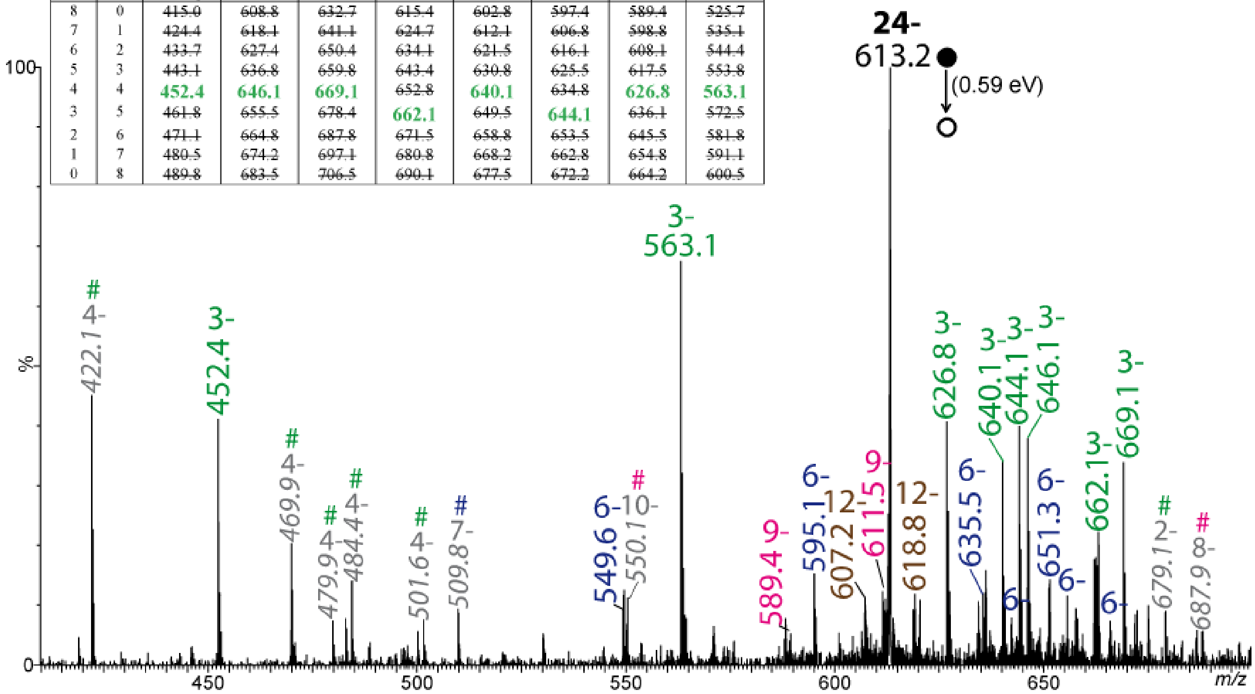

Supplementary Figure 21. Sequencing of a 8-bytes polymer that contains the ASCII-encoded word Sequence (Supplementary Table 1, Entry 11). (**a**) Negative ion mode ESI mass spectrum (MS[1]). Bold numbers represent the different charge states observed for the polymer. Grey diamonds and grey squares indicate in-source fragments and synthesis impurities, respectively. (**b**) ESI-MS[2] spectrum of the $[M\text{-}24H]^{24-}$ precursor ion at *m/z* 613.2, where *m/z* values measured for triply charged byte-fragments (in green) reveal both their **0**/**1** composition and their initial location in the polymeric chain (see inset table). Other fragment assignment is indicated in Supplementary Figure 22. These data were recorded at a 0.59 eV collision energy (center-of-mass frame) during 1 min (*i.e.*, 57 scan).

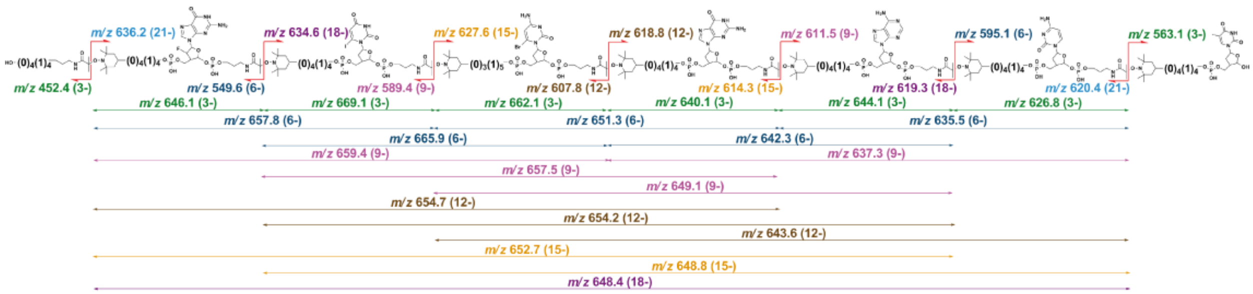

Supplementary Figure 22. Sequencing of a 8-bytes polymer that contains the ASCII-encoded word Sequence (Supplementary Table 1, Entry 11). Dissociation scheme for assignment of major fragments detected in the MS^2 spectrum of the $[M-24H]^{24-}$ precursor ion at *m/z* 613.2 shown in Supplementary Figure 21b.

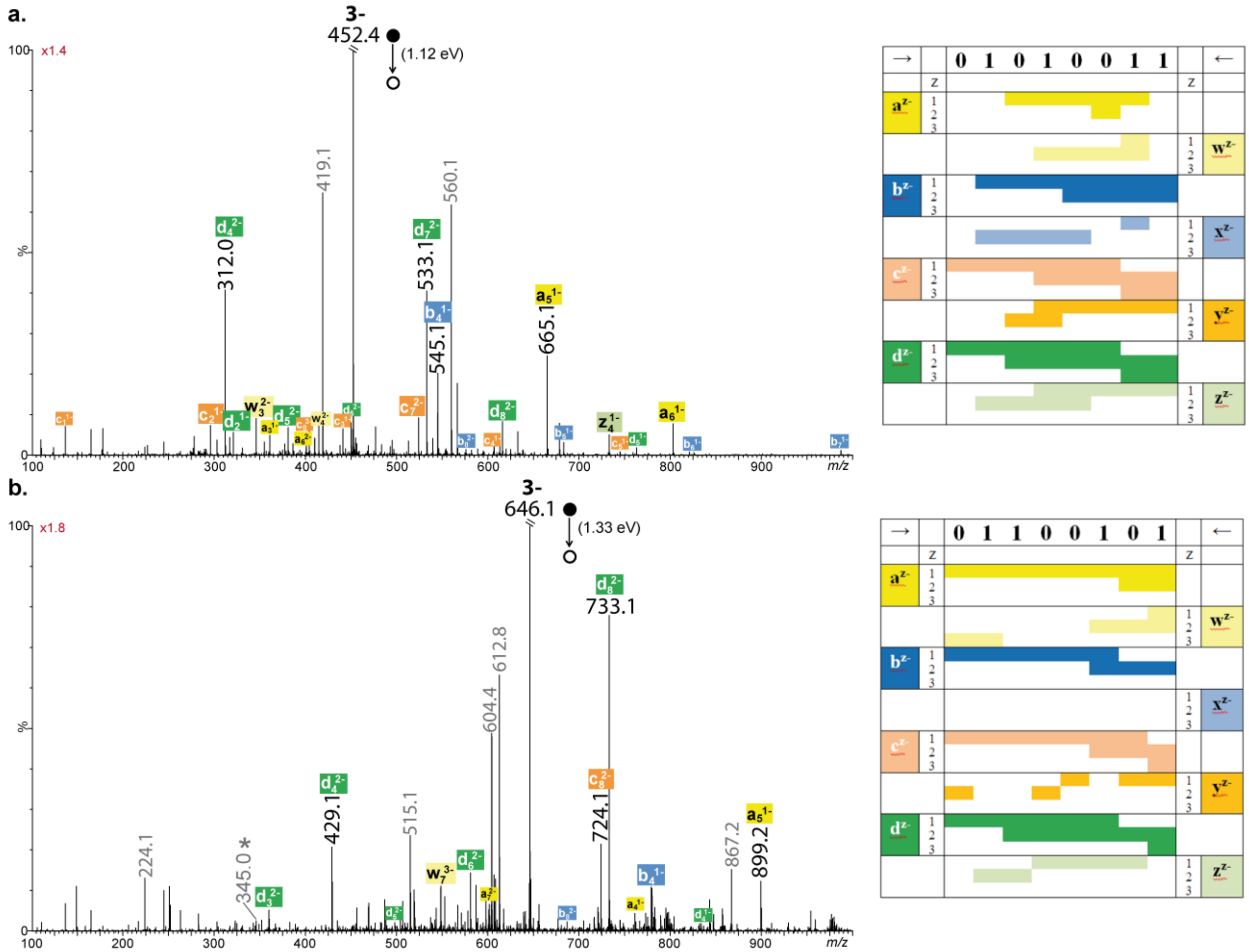

Supplementary Figure 23. Sequencing of a 8-bytes polymer that contains the ASCII-encoded word Sequence (Supplementary Table 1, Entry 11). Pseudo-MS^3 spectra (left) and associated sequence coverage (right) for (**a**) the *m/z* 452.4 fragment containing a $(\mathbf{0})_4(\mathbf{1})_4$ byte holding no tag, consistent with the expected **01010011** sequence of the 1st byte, and (**b**) the *m/z* 646.1 fragment containing a $(\mathbf{0})_4(\mathbf{1})_4$ byte holding tag F, consistent with the expected **01100101** sequence of the 2nd byte. Peaks annotated in grey correspond to products formed during reactions induced by the carbon-centered radical, with those designated by an asterisk being diagnostic of the tagging base (see Supplementary Figure 39). These data were recorded during 3 min (*i.e.*, 174 scan) using collision energies as indicated in the center-of-mass frame.

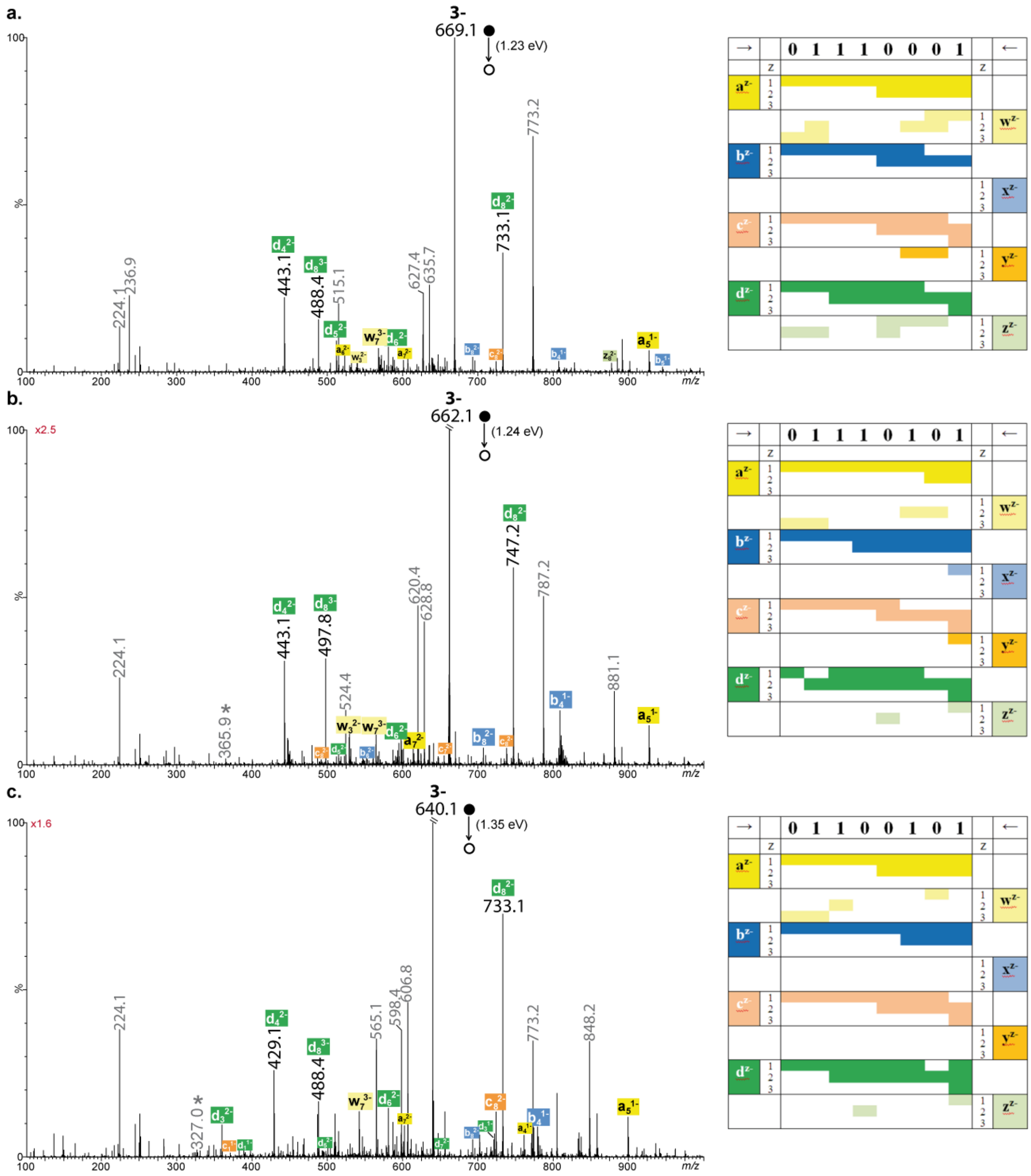

Supplementary Figure 24. Sequencing of a 8-bytes polymer that contains the ASCII-encoded word Sequence (Supplementary Table 1, Entry 11). Pseudo-MS^3 spectra (left) and associated sequence coverage (right) for (**a**) the *m/z* 669.1 fragment containing a $(\mathbf{0})_4(\mathbf{1})_4$ byte holding tag I, consistent with the expected **01110001** sequence of the 3rd byte, (**b**) the *m/z* 662.1 fragment containing a $(\mathbf{0})_3(\mathbf{1})_5$ byte holding tag B, consistent with the expected **01110101** sequence of the 4th byte, and (**c**) the *m/z* 640.1 fragment containing a $(\mathbf{0})_4(\mathbf{1})_4$ byte holding tag G, consistent with the expected **01100101** sequence of the 5th byte. Peaks annotated in grey correspond to products formed during reactions induced by the carbon-centered radical, with those designated by an asterisk being diagnostic of the tagging base (see Supplementary Figure 39). These data were recorded during 3 min (*i.e.*, 174 scan) using collision energies as indicated in the center-of-mass frame.

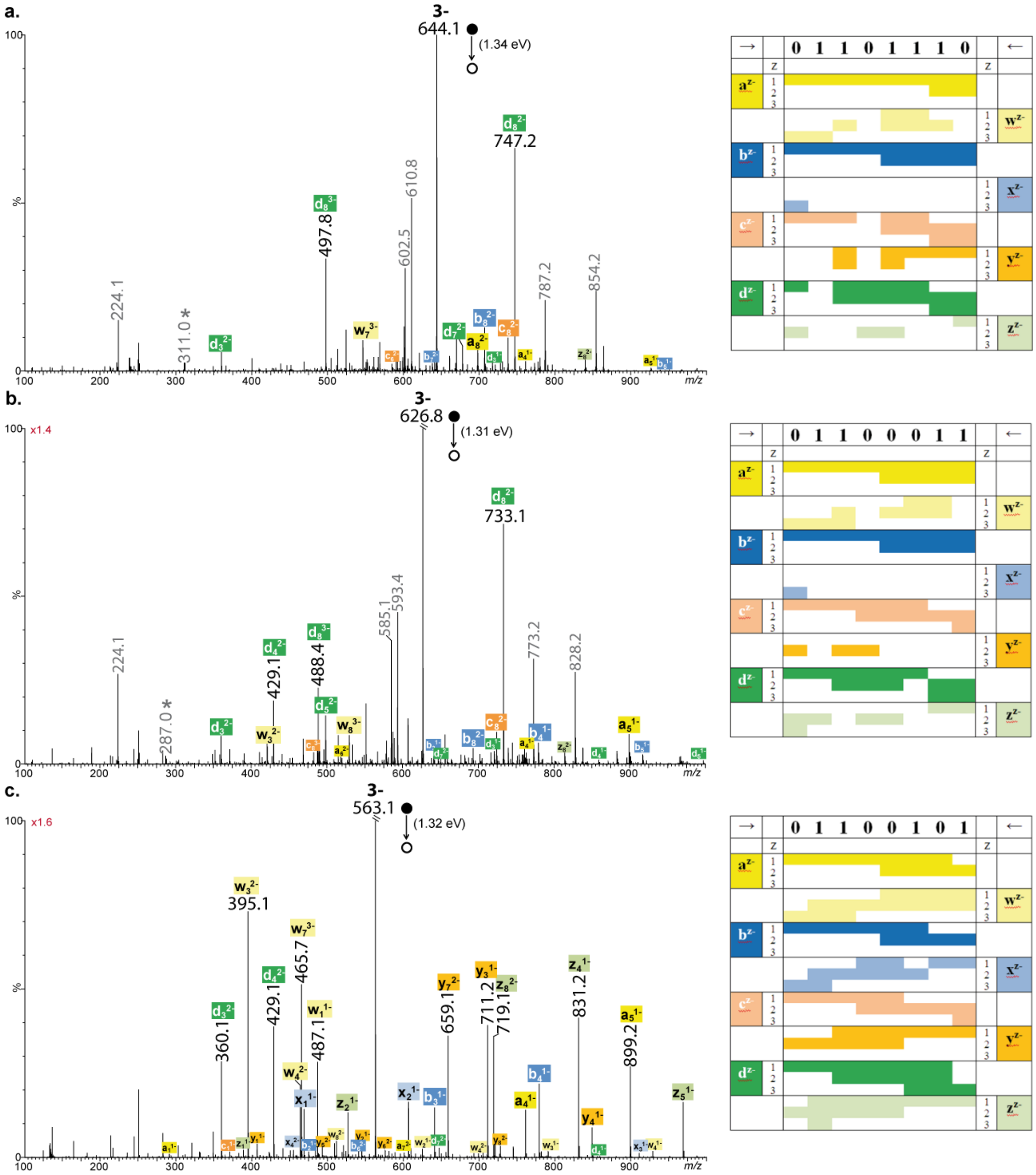

Supplementary Figure 25. Sequencing of a 8-bytes polymer that contains the ASCII-encoded word Sequence (Supplementary Table 1, Entry 11). Pseudo-MS^3 spectra (left) and associated sequence coverage (right) for (**a**) the *m/z* 644.1 fragment containing a $(\mathbf{0})_3(\mathbf{1})_5$ byte holding tag A, consistent with the expected **01101110** sequence of the 6th byte, (**b**) the *m/z* 626.8 fragment containing a $(\mathbf{0})_4(\mathbf{1})_4$ byte holding tag C, consistent with the expected **01100011** sequence of the 7th byte, and (**c**) the *m/z* 563.1 fragment containing a $(\mathbf{0})_4(\mathbf{1})_4$ byte holding tag T, consistent with the expected **01100101** sequence of the 8th byte. Peaks annotated in grey correspond to products formed during reactions induced by the carbon-centered radical, with those designated by an asterisk being diagnostic of the tagging base (see Supplementary Figure 39). These data were recorded during 3 min (*i.e.*, 174 scan) using collision energies as indicated in the center-of-mass frame.

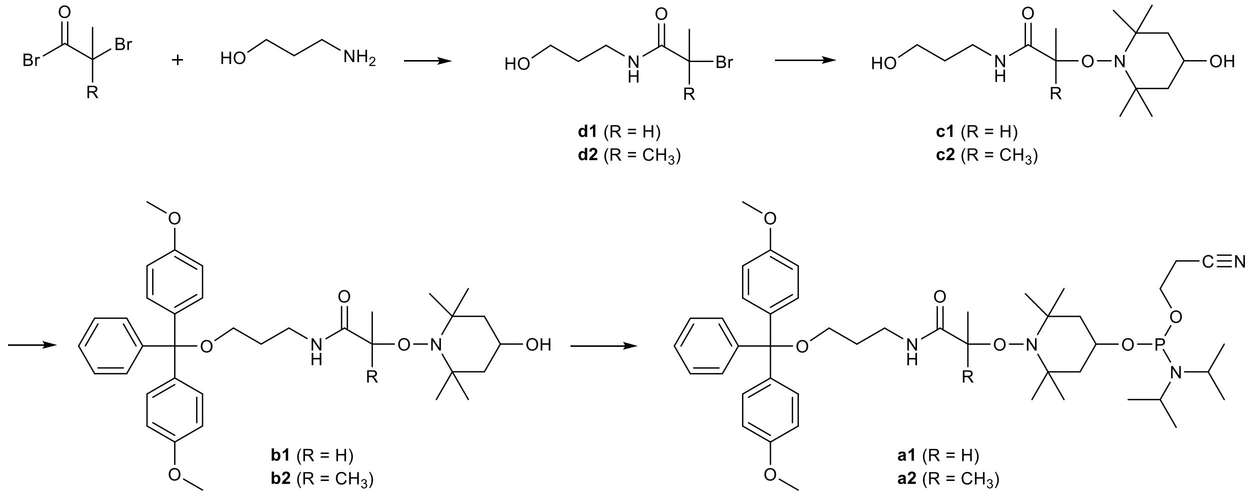

Supplementary Figure 26. Strategy used for the synthesis of the alkoxyamine-containing monomers **a1** and **a2**.

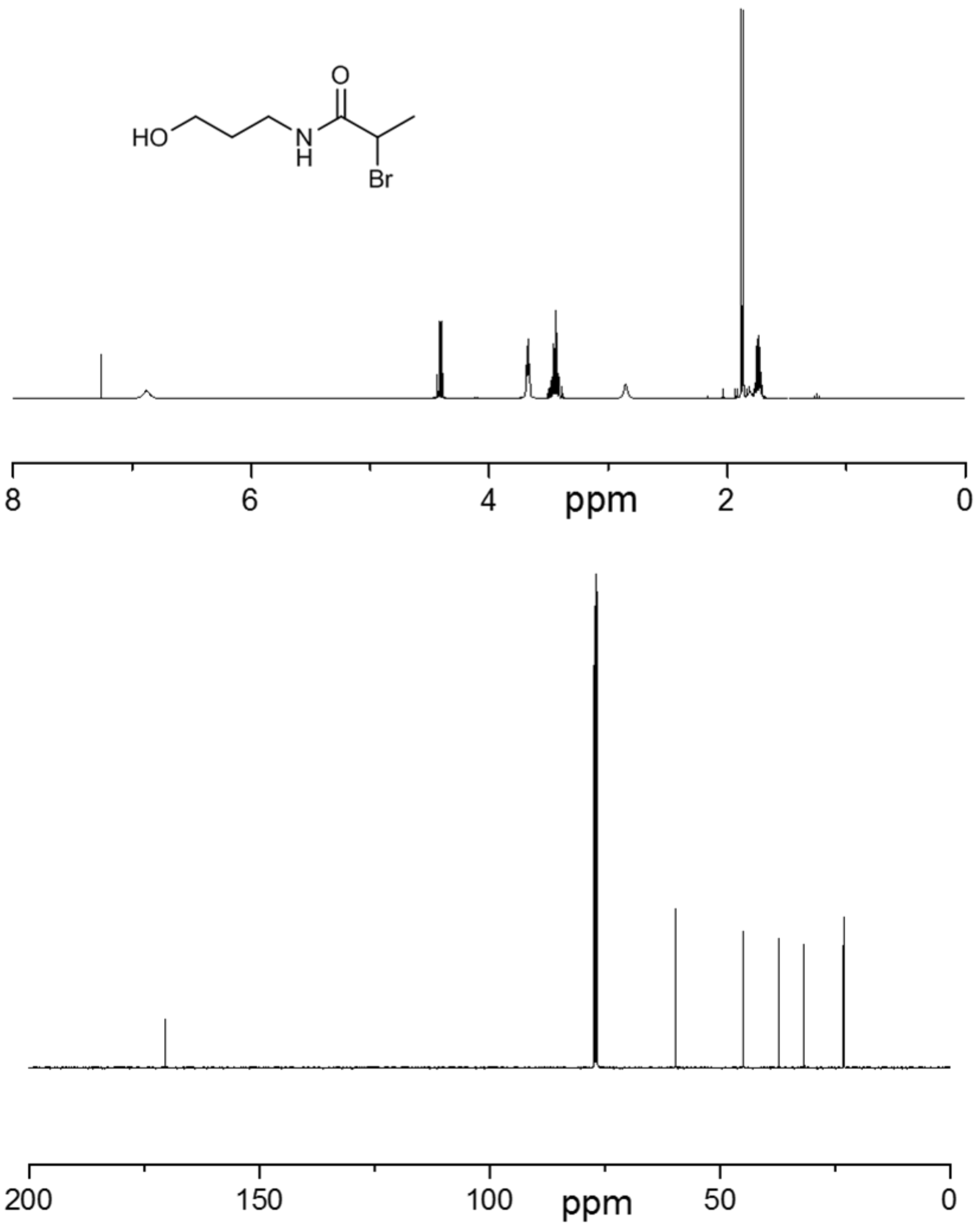

Supplementary Figure 27. ^{1}H and ^{13}C NMR spectra of **d1**. See methods section for peaks assignments.

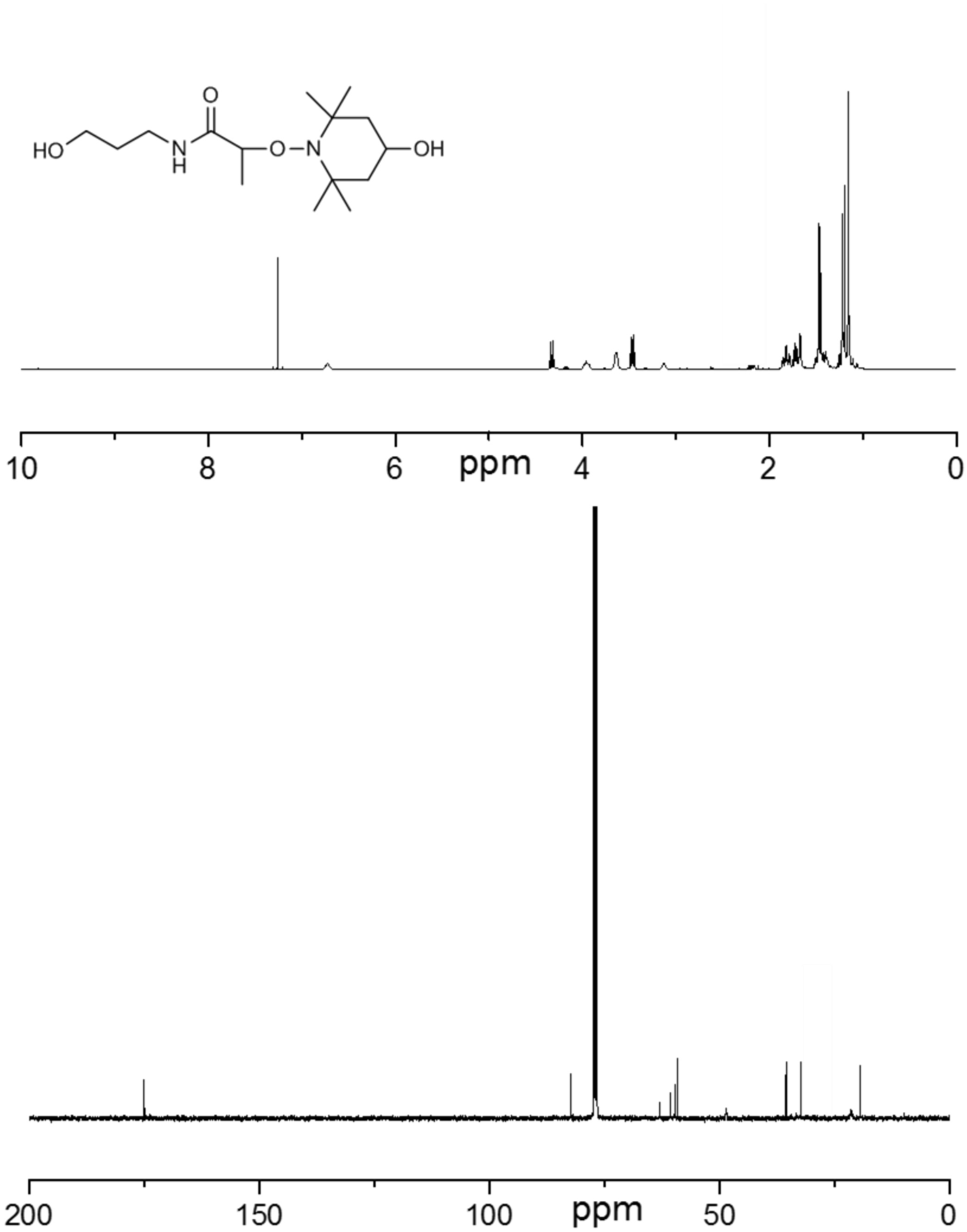

Supplementary Figure 28. ^{1}H and ^{13}C NMR spectra of **c1**. See methods section for peaks assignments.

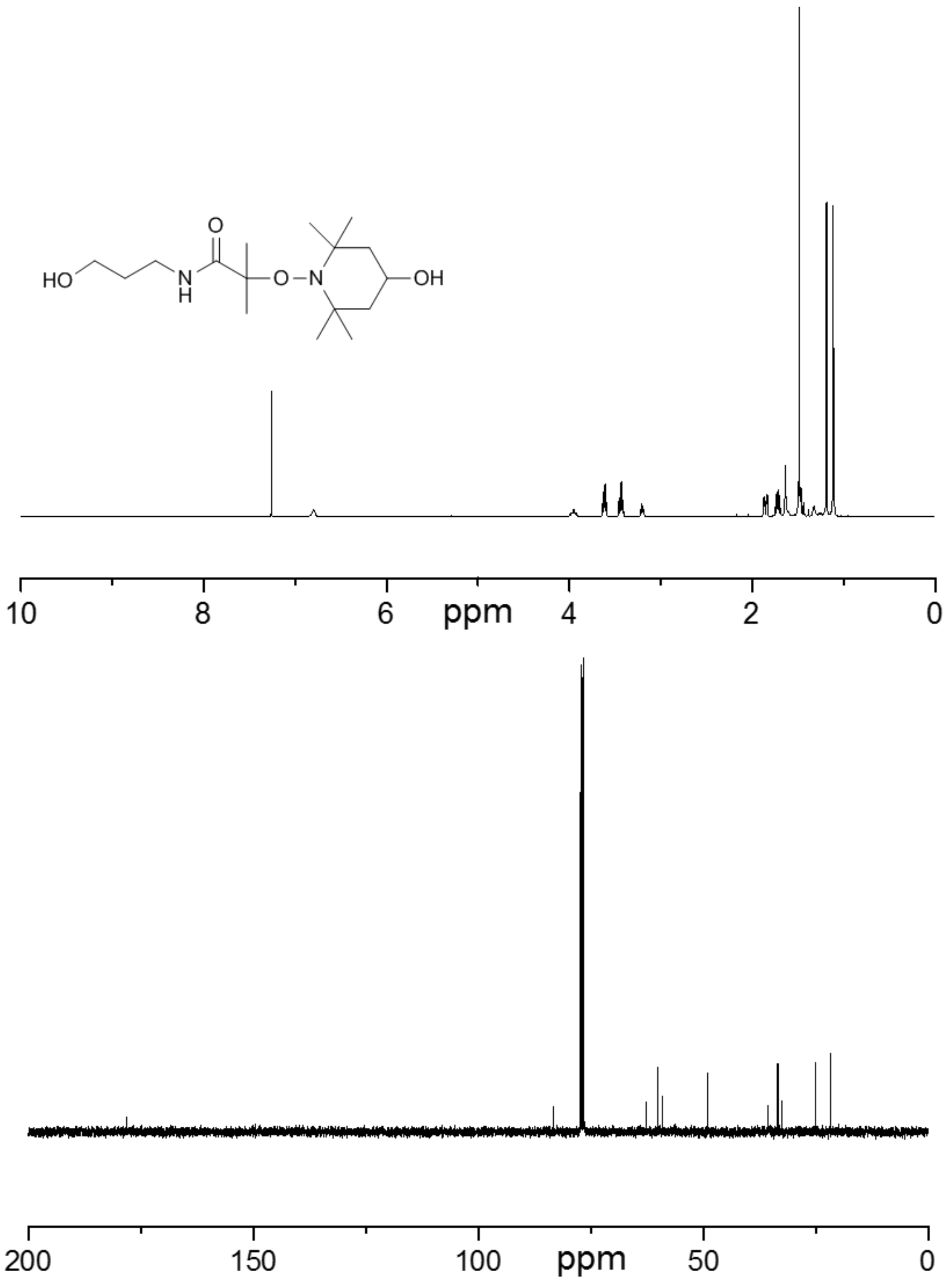

Supplementary Figure 29. ^{1}H and ^{13}C NMR spectra of **c2**. See methods section for peaks assignments.

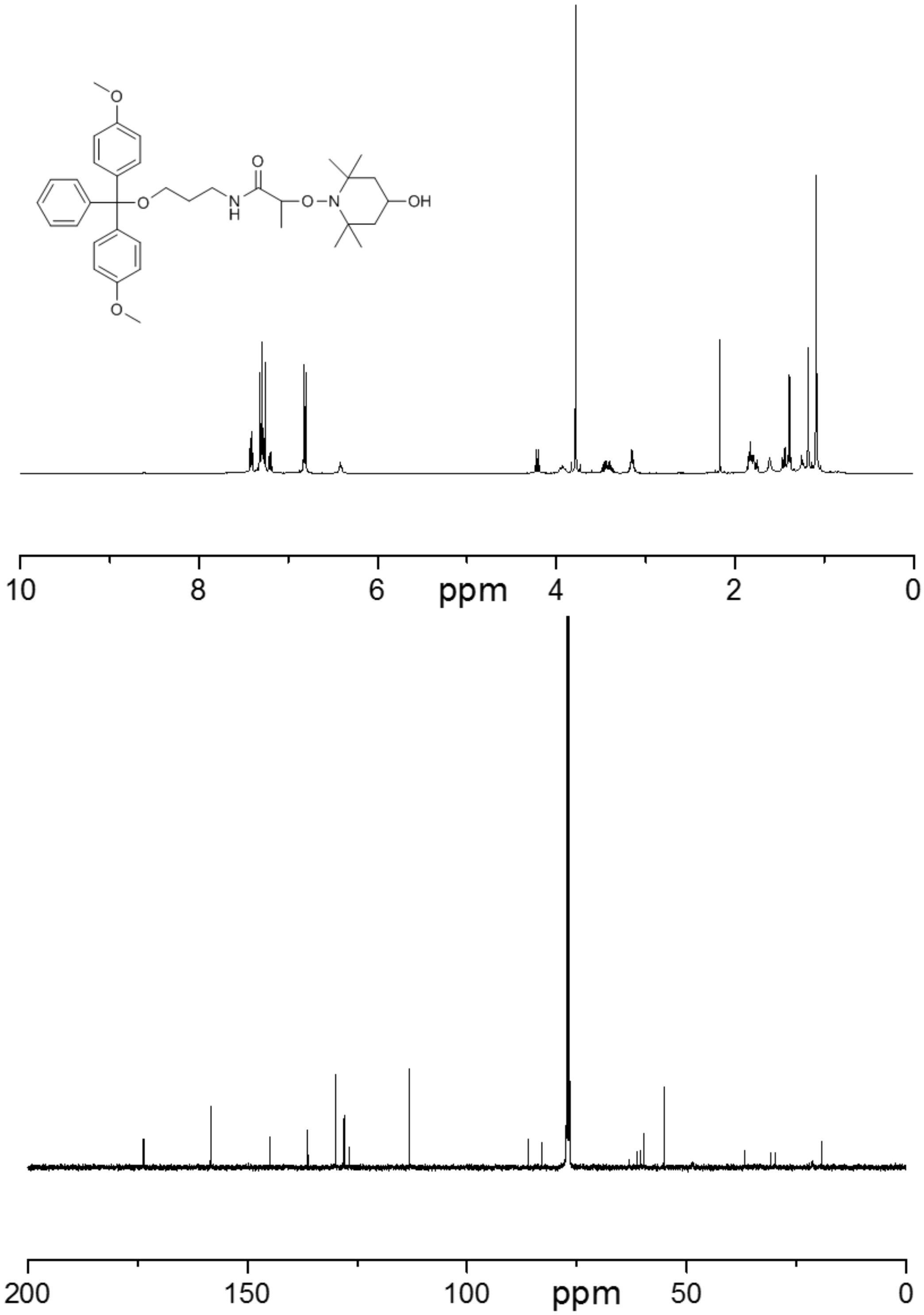

Supplementary Figure 30. ^{1}H and ^{13}C NMR spectra of **b1**. See methods section for peaks assignments.

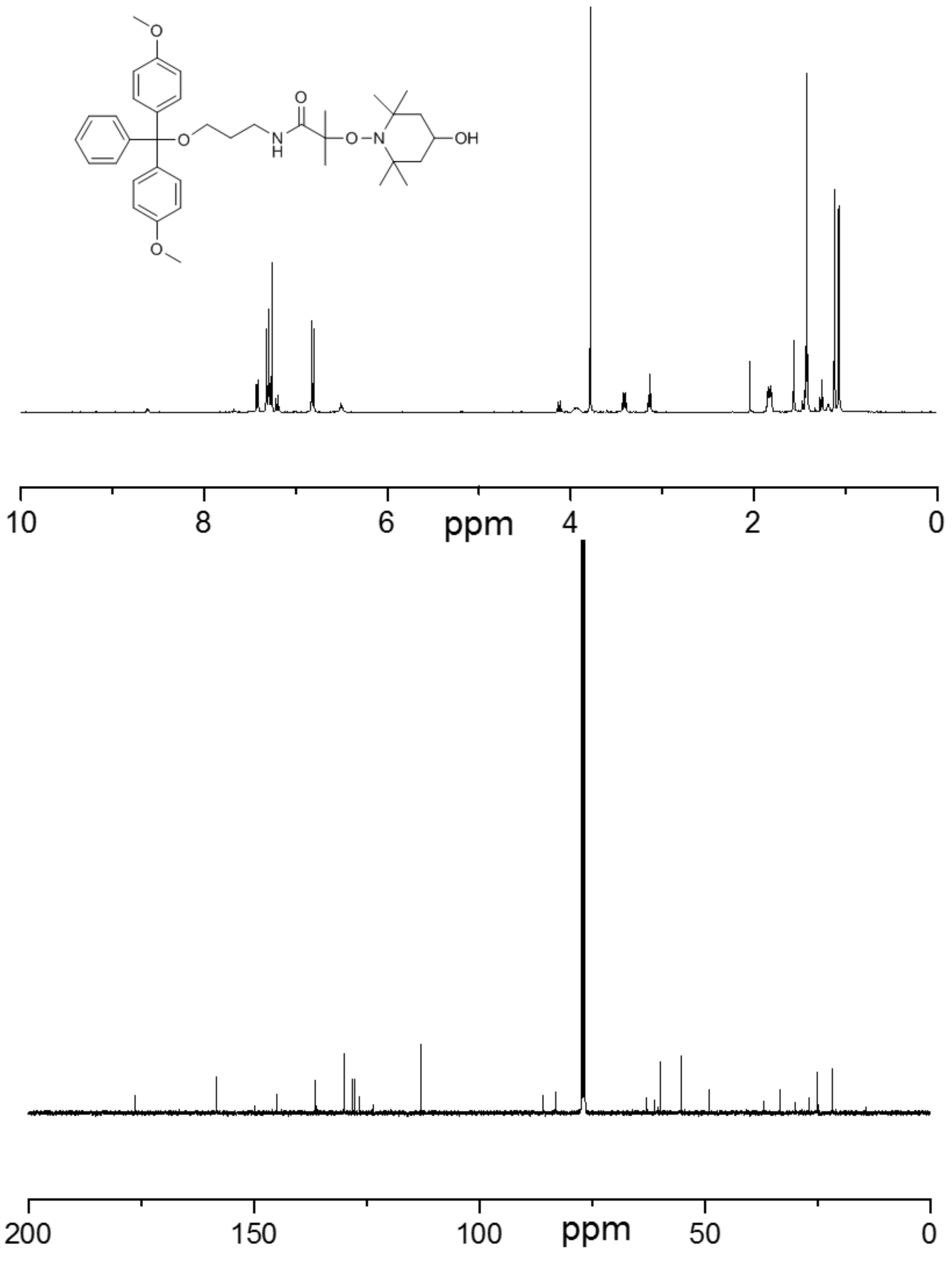

Supplementary Figure 31. ^{1}H and ^{13}C NMR spectra of **b2**. See methods section for peaks assignments.

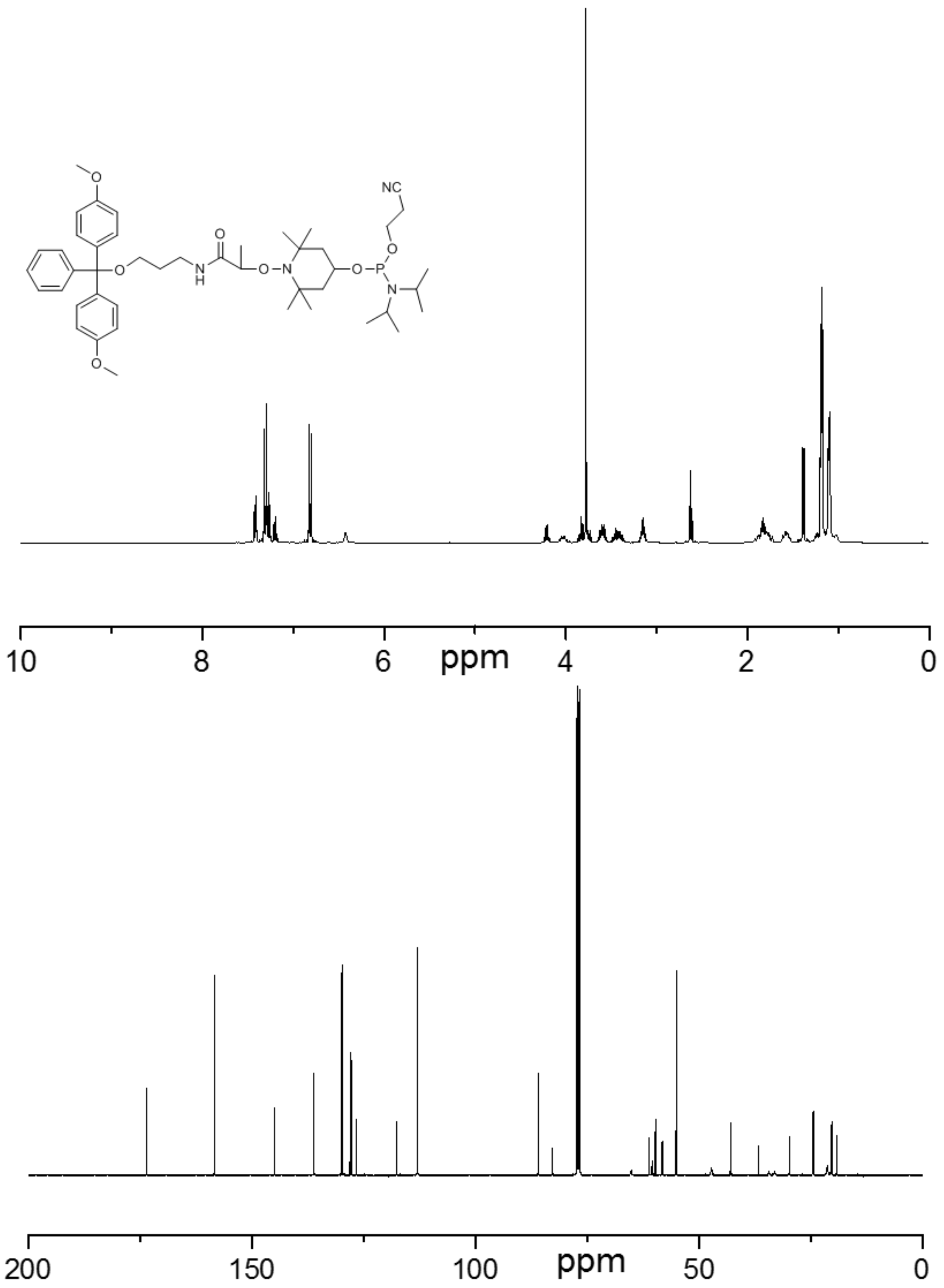

Supplementary Figure 32. ^{1}H and ^{13}C NMR spectra of **a1**. See methods section for peaks assignments.

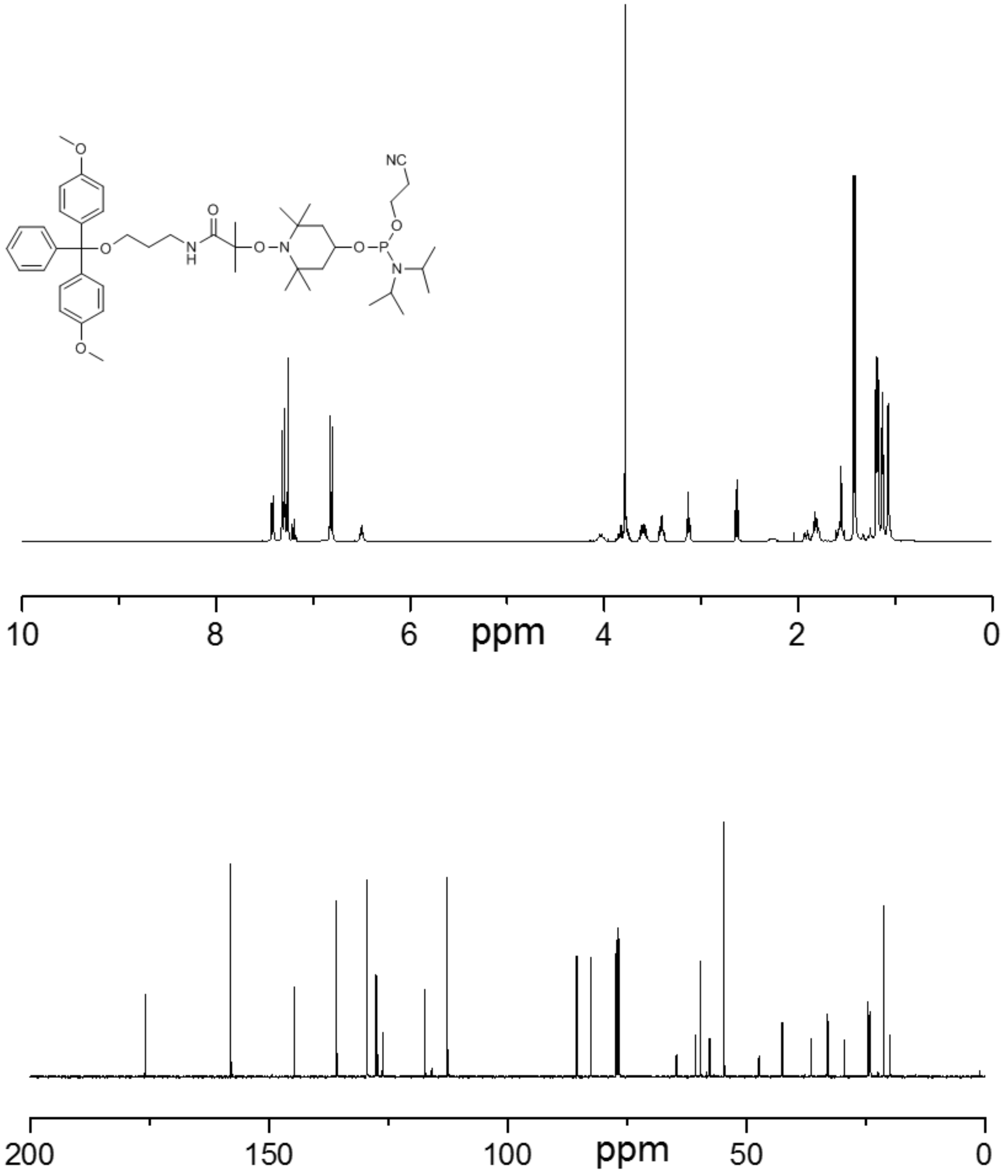

Supplementary Figure 33. ^{1}H and ^{13}C NMR spectra of **a2**. See methods section for peaks assignments.

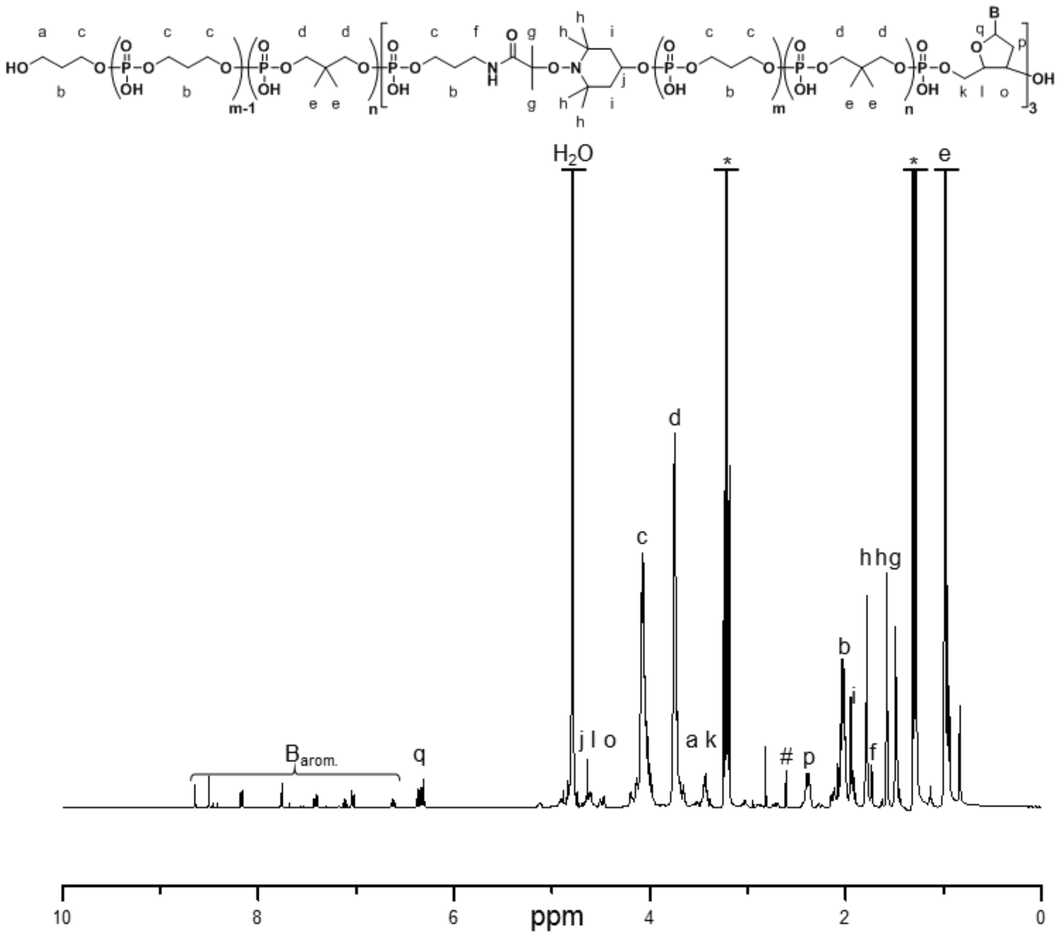

Supplementary Figure 34. ^{1}H NMR spectrum of a 4-bytes polymer that contains the ASCII-encoded word "Code" (Supplementary Table 1, Entry 7) in D_2O. The aromatic protons of the nucleobases were regrouped under $B_{arom.}$, (*) triethylammonium cations as counterions, (#) methylamine.

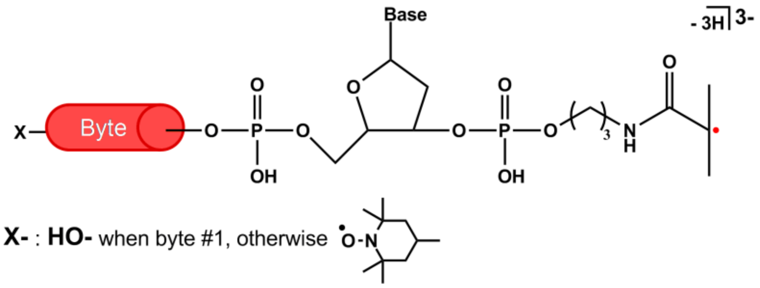

Supplementary Figure 35. Structure of primary fragments, containing any byte-segment but the last, released in MS^2 experiments upon alkoxyamine bond homolysis and hence containing a carbon-centered radical (in red) as their right-hand side termination.

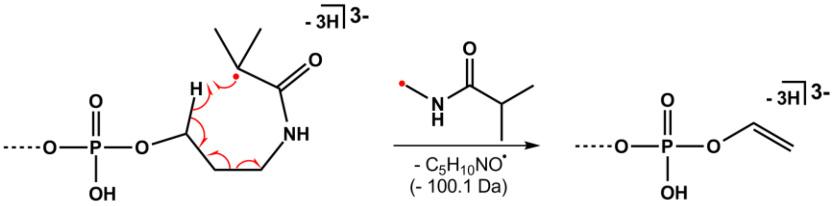

Supplementary Figure 36. Proposed mechanism for the loss of a 100.1 Da radical from all byte-fragment holding a carbon-centered radical in the ω-termination.

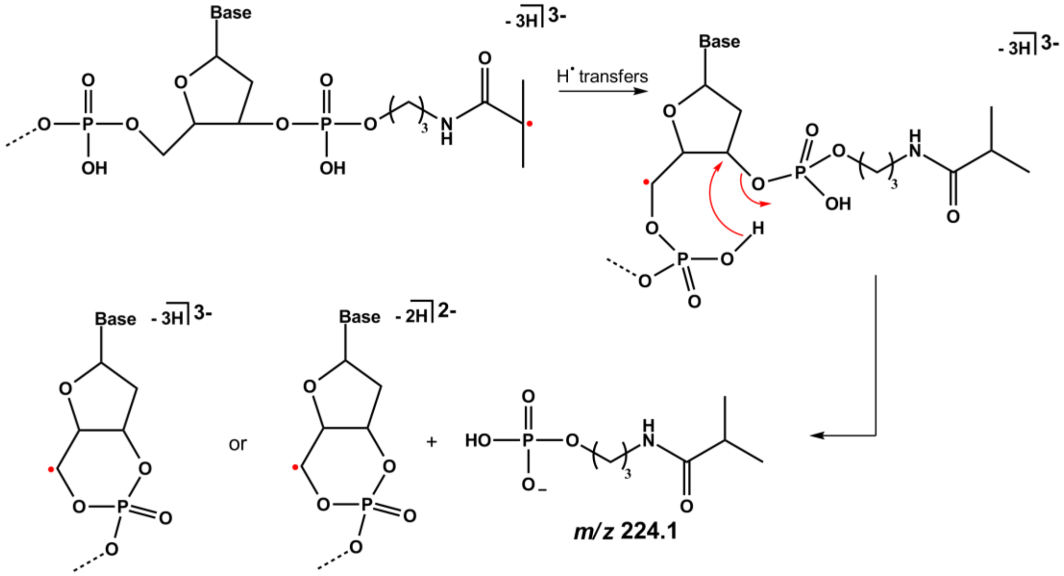

Supplementary Figure 37. Proposed mechanism for the loss of a 225.1 neutral complementarily to the production of the *m/z* 224.1 product ion from all byte-fragment holding a carbon-centered radical in the ω-termination.

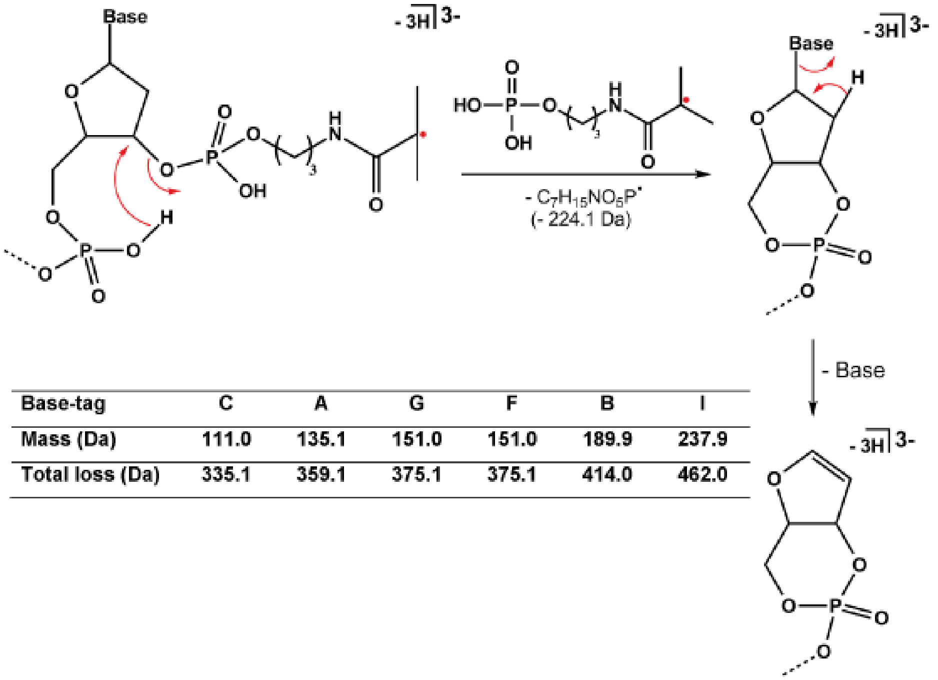

Base-tag	C	A	G	F	B	I
Mass (Da)	111.0	135.1	151.0	151.0	189.9	237.9
Total loss (Da)	335.1	359.1	375.1	375.1	414.0	462.0

Supplementary Figure 38. Proposed mechanism for the combined loss of a 224.1 Da radical and the base from base-tagged byte-fragment holding a carbon-centered radical in the ω-termination.

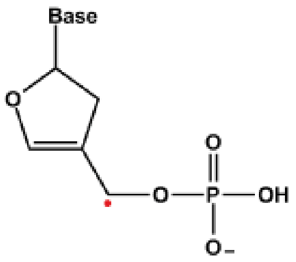

Base-tag	C	A	G	F	B	I
m/z	287.0	311.0	327.0	345.0	365.9	413.9

Supplementary Figure 39. Structure and *m/z* values of the tag-containing product ion generated from base-tagged byte-fragment holding a carbon-centered radical in the ω-termination.

Supplementary Table 1. Digital polymers synthesized and sequenced in the present work.

	**Sequence**	**m_{iso} (mg)**	**Yield %**
**1**	01000011-01001110-01010010-01010011T	3.7	66%
**2**	ATAATAAT-ATAAAATT-ATATAATT	6.1	80%
**3**	01001000-a1-01101001T	2.9	90%
**4**	01001000-a2-01101001T	3.0	92%
**5**	01000010-a2-01111001A-a2-01110100C-a2-01100101T	4.4	59%
**6**	01110100-a2-01110100A-a2-01110100C-a2-01110100T	4.7	63%
**7**	01000011-a2-01101111A-a2-01100100C-a2-01100101T	4.5	60%
**8**	01001111-a2-01100011G-a2-01110100A-a2-01100101C-a2-01110100T	5.0	53%
**9**	01000100-a2-01101001G-a2-01100111A-a2-01101001C-a2-01110100T	4.7	49%
**10**	01000010-a2-01101001B-a2-01101110G-a2-01100001A-a2-01110010C-a2-01111001T	4.9	42%
**11**	01010011-a2-01100101F-a2-01110001I-a2-01110101CB-a2-01100101G-a2-01101110A-a2-01100011C-a2-01100101T	5.6	35%

m_{iso} = isolated amount in mg after purification, the yield is based on the CPG loading (1 μmole column, 33 mM/g)

Supplementary Table 2. Assignment of in-source fragments observed in the negative mode ESI-MS shown in Figure 2a for the 4-byte digital polymer that contains the ASCII-encoded word "Byte" (Supplementary Table 1, Entry 5).

assignment	elemental composition	m/z_{th}	m/z_{exp}
[byte #1 + byte #2]$^{6-}$	$C_{95}H_{191}N_8O_{77}P_{18}^{6-}$	539.1103	539.1104
[byte #4]$^{3-}$	$C_{51}H_{100}N_3O_{41}P_9^{3-}$	563.1162	563.1183
[byte #3 + byte #4]$^{6-}$	$C_{108}H_{212}N_8O_{85}P_{19}^{6-}$	595.1266	595.1257
[byte #3]$^{3-}$	$C_{57}H_{112}N_5O_{44}P_{10}^{3-}$	626.8024	626.8013
[byte #2]$^{3-}$	$C_{60}H_{116}N_7O_{43}P_{10}^{3-}$	644.1499	644.1483
[byte #1]$^{2-}$	$C_{35}H_{76}NO_{34}P_8^{2-}$	651.1080	651.1069

Supplementary Table 3. Accurate *m/z* value of triply charged fragments released upon inter-byte alkoxyamine bond cleavage as a function of their **0/1** composition and of the tag they hold.

**0**	**1**	"no tag"	F	I	B	G	A	C	T
8	0	415.0487	608.7596	632.7209	615.3975	602.7627	597.4311	589.4274	525.7412
7	1	424.3925	618.1034	641.0647	624.7413	612.1065	606.7749	598.7711	535.0849
6	2	433.7362	627.4471	650.4084	634.0851	621.4503	616.1186	608.1149	544.4287
5	3	443.0800	636.7909	659.7522	643.4288	630.7940	625.4624	617.4587	553.7725
4	4	452.4238	646.1347	669.0960	652.7726	640.1378	634.8062	626.8024	563.1162
3	5	461.7675	655.4784	678.4397	662.1164	649.4816	644.1499	636.1462	572.4600
2	6	471.1113	664.8222	687.7835	671.4601	658.8253	653.4937	645.4900	581.8038
1	7	480.4551	674.1660	697.1273	680.8039	668.1691	662.8375	654.8337	591.1475
0	8	489.7988	683.5097	706.4710	690.1477	677.5129	672.1812	664.1775	600.4913
